# Professional Skill Builder: Mastering Cardiac Auscultation in Under 4 Hours

**DOI:** 10.15766/mep_2374-8265.10577

**Published:** 2017-05-08

**Authors:** Rajesh Mangrulkar, Richard Judge, Chris Chapman, Cyril Grum, Peter Hagan, John Westfall, Marc Stephens, Aki Yao, Jason Engling

**Affiliations:** 1 Marguerite S. Roll Professor of Medical Education, University of Michigan Medical School; 2 Associate Dean for Medical Student Education, University of Michigan Medical School; 3 Associate Professor of Internal Medicine and Learning Health Sciences, University of Michigan Medical School; 4 Emeritus Clinical Professor of Internal Medicine, Division of Cardiology, University of Michigan Medical School; 5 Assistant Director for Learning Design and Publishing, Health Information Technology and Services, University of Michigan Medical School; 6 Professor of Internal Medicine, University of Michigan Medical School; 7 Senior Associate Chair for Undergraduate Medical Programs, University of Michigan Medical School; 8 Assistant Professor of Internal Medicine, University of Michigan Medical School; 9 Director of the Cardiovascular Disease Fellowship Program, University of Michigan Medical School; 10 Co-Director of the M2 Cardiovascular Disease Sequence, University of Michigan Medical School; 11 Multimedia Developer and Instructional Designer, Health Information Technology and Services, University of Michigan Medical School; 12 Graphic Designer, Health Information Technology and Services, University of Michigan Medical School

**Keywords:** Editor's Choice, Physical Exam, Heart Sounds, Murmurs, Auscultation, Education, Cardiac, Computer-Aided Instruction, Multimedia, Case-Based Learning, Clinical Reasoning

## Abstract

**Introduction:**

Cardiac auscultation is an important clinical skill used by health care professionals during bedside patient evaluation and management. To support development of this skill in health sciences students, we created a self-paced, interactive program. This program helps develop foundational skills and knowledge so learners can confidently perform basic cardiac auscultation at the bedside.

**Methods:**

For novice learners, this program should be used in conjunction with their initial clinical experiences so they can immediately apply what they have learned in the short course. Advanced learners and health care professionals can use this program to review and improve their cardiac auscultation skills. To achieve these objectives, this multimedia program teaches the eight basic heart cadences and their clinical significance through the use of guided tutorials, a gamified e-learning activity, interactive clinical cases, and a self-assessment. A heart sound and murmur library is also included for comparative listening at the bedside.

**Results:**

Course evaluations from the first- and second-year Clinical Foundations of Medicine courses at the University of Michigan Medical School demonstrate the value of the various sections of the program. Additionally, the clinical cases have been shown to be effective in improving cardiac auscultation knowledge and skills among residents.

**Discussion:**

All clinical cases in the program are based on authentic clinical problems and were developed by academic cardiologists and internists with expertise in this area. Various sections of this tutorial have been in use at our institution for over 20 years and have been evaluated favorably by our students.

## Educational Objectives

By the end of the module, learners will be able to:

1. Accurately identify the different heart sound cadences.
2. Describe the physiologic characteristics of the different heart sounds and cadences.
3. Interpret the clinical significance of the different heart sounds and cadences.

## Introduction

This Professional Skill Builder (PSB) allows health science students the opportunity to practice cardiac auscultation skills using simulated cases with expert faculty feedback. The PSB was originally developed in 1990 to address the growing need for training in cardiac auscultation skills. Since then, we have used the PSB with medical students in internal medicine clerkships, systems-based courses, and introductory clinical skills courses.

As an important component of the physical examination, these skills are relevant across the continuum of medical education and critical to the diagnosis of many health conditions, including mitral valve prolapse (MVP), aortic stenosis, and congestive heart failure. For the novice learner, this program is ideally used in conjunction with other curricular instruction and clinical experiences.^1^

The PSB uses audiovisual modalities, guided tutorials, a gamified e-learning activity, interactive clinical cases, and a self-assessment to facilitate learning. The program was designed in accordance with evidence-based multimedia design principles to maximize learning by reducing extraneous cognitive load, optimizing intrinsic processing, and supporting generative processing (Table 1).^2^

**Table 1.** Multimedia Design Principles and Their Application in the Professional Skill Builder

Evidence-Based Multimedia Design Principle	Description	Application
Reduce extraneous cognitive load	Processing that does not support the learning objectives (e.g., too much information, fun and interesting but irrelevant material, poor user experience design).	The information presented pertains directly to the learning objectives. We eliminated nonessential material, highlighted lesson organization, and provided an easy-to-use interface.
Optimize essential processing	Processing that helps learners remember and understand information and form cognitive models of the material. Essential processing is enhanced by using spoken words (vs. text), segmenting content into chunks, and explaining concepts and terms before using them in instruction.	In the Basics of Cardiac Auscultation and the Heart Sound Primer, audio narration accompanies still and moving images; concepts and terms used in other parts of the program are explained. The entire program is chunked into modules and screens.
Promote generative processing	Processing that allows learners to apply their knowledge to real-life problems and situations. Generative processing is facilitated by using words in combination with relevant pictures and by using a conversational tone and style in speech and writing.	A conversational, personalized tone and words with relevant pictures are used throughout the program.

To promote and deepen the learner's critical thinking, the cases in the Clinical Cases section were designed using a Socratic approach.^3^ The cases are structured so that the learner is presented with a logical, goal-directed sequence of questions with expert feedback to guide him or her through the clinical reasoning process. This instructional approach allows learning to be more efficient and effective, as well as providing a means of engagement between the learner and the faculty expert.

The PSB's integration of tutorial-based instruction, gamified e-learning, and Socratic instruction is different from other programs covering this content. The others focus on high-fidelity visualizations (e.g., Blaufuss Multimedia Laboratories), mannequin simulation technologies (e.g., Harvey—the Cardiopulmonary Patient Simulator), and audio sound banks (e.g., Auscultation Skills: Breath & Heart Sounds).

## Methods

The appropriate audience for this educational tool is broad and includes early and intermediate learners who are developing their communication, data-gathering, fundamental physical exam, and decision-making skills. PSB learners include medical, nursing, dental, and physician assistant students. A basic understanding of cardiovascular physiology is required before beginning this tutorial, usually obtained in any introductory level physiology college course. Fundamental objectives^4^ that should be achieved prior to initiating this module include the following:

• Understanding the basic functional anatomy of the atrioventricular and semilunar valves and how they operate.
• Understanding the pressure, volume, heart sound, and changes in the cardiac cycle.
• Identifying the intervals of contraction, ejection, relaxation, and filling in both isovolumic and dynamic volumic states.
• Understanding the phases of ventricular systole and ventricular diastole.
• Contrasting the relationship between pressure and flow into and out of the left and right ventricles during each phase of the cardiac cycle.
• Understanding how and why left-sided and right-sided events differ in their timing.
• Knowing the factors that contribute to the formation of turbulent flow.
• Describing the timing and causes of the four heart sounds and the expected auscultation sounds that define mitral stenosis, mitral insufficiency, aortic stenosis, and aortic insufficiency.
• Explaining how pathologic changes to valvular function affect cardiac mechanics and blood pressure.

Residents, fellows, and primary care physicians may also benefit from regular refreshers of these skills at times or as needed.

In order to run this module on your computer, you must have about 170 MB of available space. Your computer must run the following operating systems in order to view the module: OSX for a Mac, iOS 7 or higher for Apple mobile products, and Windows 7 and above for PC. It is recommended that you use the Chrome, Safari, or Firefox browsers on a Mac, or Chrome and Firefox on a Windows PC. Additionally, headphones or earbuds are recommended when listening to the heart and breath sounds.

The PSB Mastering Cardiac Auscultation folder (Appendix A) contains all the necessary files to run the PSB. Download the folder (a zip file) onto your computer by clicking on the Download button on the MedEdPORTAL page. Once you have downloaded the file onto your computer, unzip the file to make the program available for use. For Mac users, double-click on the downloaded file. For PC users, press and hold (or right-click) the folder, select Extract All, and then follow the instructions. Once you have unzipped the file and placed it on your desktop, locate the program folder entitled psb-mastering-cardiac-auscultation on your computer. Open the index.html file in the folder with one of the recommended internet browsers. The PSB home page will display the program in the Chrome window. Use the PSB menu to select and view the program contents.

### Basics of Cardiac Auscultation (10 Minutes)

This section provides the learner with an orientation to the use of the stethoscope and technique of cardiac auscultation, as well as the interpretation of the diagrams used to depict the various normal and abnormal cardiac cadences. Video vignettes employing narration, animation, heart sounds, and phonocardiograms are used in combination to introduce and compare the sounds and cadences. Finally, the use of poetic meter is correlated with the cadences to help the learner anchor the cadences in memory.

### Heart Sound Primer (12 Minutes)

This section systematically presents the eight basic heart sound cadences and their corresponding location to the learner:

1. Apex area, normal S_1_ S_2_.
2. Tricuspid area, split S_1_.
3. Pulmonic area, split S_2_.
4. Aortic area, S_1_ S_2_.
5. Aortic area, systolic murmur.
6. Aortic area, diastolic murmur.
7. Apex area, S_3_ gallop.
8. Apex area, S_4_ gallop.

Animation, narration, and illustrations are used to provide the learner with a breakdown of each composite sound in a particular cadence along with a corresponding physiologic explanation of its generation. In order to enhance learning, the user is offered the option to use his/her own stethoscope to listen to the four normal cadences on his/her own body.

### Heart Sound Challenge (45–90 Minutes)

In this section, a gamelike activity allows the user to practice listening and identifying the cardiac cadences until he/she commits them to automatic recognition. The structure of the activity provides an incentive for the learner to practice his/her listening skills without the boredom typically associated with the amount of repetitive practice that has been shown to be required for rapid, automatic recognition of a cadence. In this exercise, the eight cadences are presented randomly, and the learner is required to correctly identify each. The challenge is to correctly identify 24 cadences in a row. If the learner incorrectly identifies a particular cadence, he/she has the option to see a hint (e.g., the location and stethoscope end-piece associated with the finding), as well as to listen and compare his/her selection with the cadence being identified. This allows learners to fill the gaps in their skills as they work towards the automatic recognition of the cadences.

### Clinical Cases (11 Cases, 10 Minutes per Case)

Eleven clinical cases are then presented to allow the learner to practice and synthesize his/her auscultation skills on authentic patient problems. In each case, the learner is provided with a presenting scenario with relevant history, physical, and social information. The learner is asked to listen to and identify an auscultatory finding. Based on the position of the patient, the location where the learner is listening, and the end-piece used, the learner is then guided through a series of questions to identify the cause of the finding and its significance. In some cases, auscultation is performed on more than one position. All cases conclude with a summary featuring key take-home points.

The topics of the cases include the following:

• Normal cardiac exam in a patient with coronary artery disease (CAD).
• Mitral regurgitation (MR) and S_4_ in a patient with CAD.
• Subacute bacterial endocarditis in a patient with chronic MR.
• Bicuspid aortic valve with aortic regurgitation in a 17-year-old athlete.
• Calcific aortic stenosis with anginal chest pain in a middle-aged police officer.
• S_4_ in a hypertensive patient.
• Systolic click in 19-year-old student with palpitation.
• S_3_ gallop in patient with an acute anteroseptal myocardial infarction.
• MVP with MR in a student with atypical chest pain.
• Split S_1_ in a woman with a family history of MVP.
• S_3_ and MR in a patient with congestive heart failure due to dilated cardiomyopathy.

### Self-Assessment (50 Minutes)

In this section, the learner is given the opportunity to reassess his/her auscultatory knowledge and skills using the same techniques employed in each of the previous sections. This self-assessment is included to provide additional practice so as to improve learner confidence and demonstrate any area needing remediation.

The self-assessment covers the accurate identification of heart sounds, the accurate identification of murmurs, the accurate identification of the optimal location of auscultatory findings with different underlying causes, and the clinical cases (to evaluate clinical knowledge and skills pertaining to cardiac auscultation).

### Heart Sound and Murmur Library

This section provides 23 common heart sounds and murmurs that can be easily and rapidly accessed. For each, the position of the patient, the location on the chest where the sound or murmur is found, and the end-piece of the stethoscope used are designated, along with the most common cause. The library is ideal for comparative listening in the clinical setting. Additionally, the audio files can be copied to mobile devices for portability and use in locations where internet connectivity is not available.

## Results

Course evaluations from both the first- and second-year Clinical Foundations of Medicine courses at the University of Michigan Medical School demonstrate the value of the various sections of the Professional Skill Builder (Table 2).

**Table 2.** Summary of First- and Second-Year Clinical Foundations of Medicine Course Evaluations

Clinical Foundations of Medicine	Item	2013-2014		2014-2015	
		*M*^a^ (*SD*)	*N*	*M*^b^ (*SD*)	*N*
First year	The Professional Skill Builder Heart Sounds Primer helped me in learning to perform the cardiac exam on a person.	4.38 (.73)	29	4.26 (.84)	136
	The Professional Skill Builder Heart Sounds Primer helped me in learning to recognize specific heart sounds and murmurs.	4.76 (.44)	29	4.52 (.66)	136
	The Professional Skill Builder Heart Sound Challenge game component helped motivate me to practice listening to heart sounds and murmurs.	4.60 (.58)	25	4.36 (.82)	134
Second year	Value of Chest Pain Professional Skill Builder exercises.	3.64 (.93)	14	3.61 (.94)	57

a Based on a 5-point scale (1 = *strongly disagree*, 5 = *strongly agree*).

b Based on a 5-point scale (1 = *poor*, 5 = *excellent*)

Students also provided narrative comments about the program.

• “Heart challenge was genius. I wouldn't have been motivated to learn the sounds any other way.”
• “The PSBs were amazingly helpful.”
• “PSBs were very helpful in terms of clinical applications.”
• “The online PSB modules were great, but I wish we could have listened to actual heart sounds rather than the computer-generated clips that were used.”
• “I really liked the PSB modules, I felt these were one of the strongest parts of the course.”
• “Loved the online modules (PSBs). These were very helpful for learning heart murmurs.”
• “The PSB was a great resource and was a very good component of the sequence.”
• “The PSB were a bit too long for the amount of help that they provided.”
• “PSB modules didn't really help me much at all. Multimedia components of Dr. [X's] lectures were very sufficient for learning.”
• “The PSB modules were fantastic. They helped me apply the knowledge I learned in class to a real life scenario. This was an exciting and important part of my learning for this sequence. I particularly think that the emphasis on developing a differential diagnosis (probable, possible, improbable, etc.) was invaluable.”

Additionally, the clinical cases that are part of this application have been shown to be effective in improving cardiac auscultation knowledge and skills among residents. In one study, residents (*N* = 127) from two institutions participating in a 1-month primary care rotation were stratified into two groups: the intervention group receiving a 20-case superset of the clinical cases module and a control group not using the resource. When compared against a pretest and posttest, cardiac auscultation skills and knowledge improved significantly in the intervention group (all < .001; Table 3).^5^

**Table 3.** Residents' Skills and Knowledge Improvement

Group	Skills		Knowledge	
	Pretest	Posttest	Pretest	Posttest
Intervention (*n* = 56)	57.60%	79.00%	64.20%	73.50%
Control (*n* = 71)	66.20%	68.50%	64.60%	66.90%

We also compared the skills of 168 third-year medical students at one medical school; those who used the PSB developed significantly greater auscultation skills than those who did not. Students were tested before and after exposure to a 20-case version of the PSB and compared to a control. Students exposed to the program showed significant improvements in auscultation skills (*p* < .05), but not knowledge, as compared to the control. After 1 year, students' auscultation skills were retained, but auscultation knowledge was not.^1^

## Discussion

The content of the various sections of this tutorial has been in use at the University of Michigan Medical School for over 20 years and has been evaluated favorably by our students. In particular, the use of heart sound recordings with the introductory explanations of their source and the repetitive listening incorporated in the Heart Sound Challenge have given students an increased degree of confidence in their auscultatory skills when the time has arrived to begin examining patients.

One notable value in introducing cardiac auscultation using computer technology is the opportunity for comparative listening it provides to the learner. This is helpful in mastering cadence recognition, and when reviewing the clinical cases, the learner in a matter of seconds can compare, for example, the systolic murmur caused by CAD with that due to MVP, or the S_4_ found in a patient with left ventricular hypertrophy due to hypertension with the S_3_ in the patient with myocardial infarction. Comparative listening, which is rarely possible at the bedside, can be a valuable way of imprinting a cadence along with its pathophysiologic basis.

Because portions of the program were developed over 20 years ago, some of the visual elements are dated (e.g., ECG tracings used throughout the program and several of the video recordings in the Basics of Cardiac Auscultation section). Although educationally sound, these media elements could possibly distract some contemporary learners used to elements with higher fidelity. Also, the course evaluation data reported in the Results section are limited to self-perception. Third, as described above, there is evidence that some of the auscultation knowledge gained can attenuate over time. We propose that learners should refresh their skills through periodic engagement with this tool or others at a more advanced level, perhaps annually, to offset the attenuation. Finally, as the program has been in continuous development since 1990, the content evaluated in the two studies presented in the Results section may be slightly different than the current version of the program.

## Appendices

A. PSB Mastering Cardiac Auscultation folder All appendices are peer reviewed as integral parts of the Original Publication.
