# Supplementary figures and images for: Professional Skill Builder: Mastering Cardiac Auscultation in Under 4 Hours
Source: MedEdPORTAL. 2017 May 8;13:10577. doi: 10.15766/mep_2374-8265.10577 (PMC6338255; doi:10.15766/mep_2374-8265.10577)

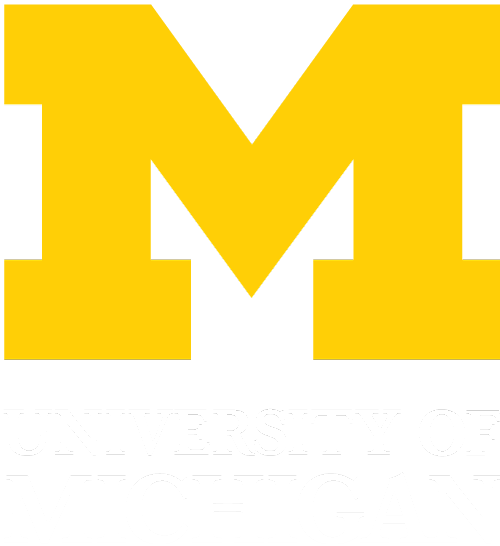

Supplement: Supplementary file 1 — A. PSB Mastering Cardiac Auscultation folder [file mep-13-10577-s001.zip › A. PSB Mastering Cardiac Auscultation folder/psb-mastering-cardiac-auscultation/assets/assets-menu/images/2color-transparentbg.png]

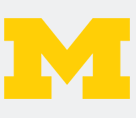

Supplement: Supplementary file 1 — A. PSB Mastering Cardiac Auscultation folder [file mep-13-10577-s001.zip › A. PSB Mastering Cardiac Auscultation folder/psb-mastering-cardiac-auscultation/assets/assets-menu/images/favicon.png]

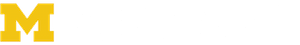

Supplement: Supplementary file 1 — A. PSB Mastering Cardiac Auscultation folder [file mep-13-10577-s001.zip › A. PSB Mastering Cardiac Auscultation folder/psb-mastering-cardiac-auscultation/assets/assets-menu/images/ummedschool.png]

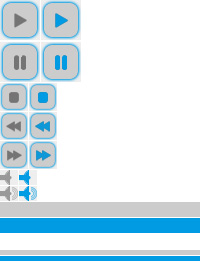

Supplement: Supplementary file 1 — A. PSB Mastering Cardiac Auscultation folder [file mep-13-10577-s001.zip › A. PSB Mastering Cardiac Auscultation folder/psb-mastering-cardiac-auscultation/assets/common/assets/css/jplayer/jplayer.blue.monday.jpg]

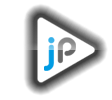

Supplement: Supplementary file 1 — A. PSB Mastering Cardiac Auscultation folder [file mep-13-10577-s001.zip › A. PSB Mastering Cardiac Auscultation folder/psb-mastering-cardiac-auscultation/assets/common/assets/css/jplayer/jplayer.blue.monday.video.play.hover.png]

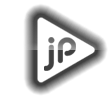

Supplement: Supplementary file 1 — A. PSB Mastering Cardiac Auscultation folder [file mep-13-10577-s001.zip › A. PSB Mastering Cardiac Auscultation folder/psb-mastering-cardiac-auscultation/assets/common/assets/css/jplayer/jplayer.blue.monday.video.play.png]

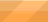

Supplement: Supplementary file 1 — A. PSB Mastering Cardiac Auscultation folder [file mep-13-10577-s001.zip › A. PSB Mastering Cardiac Auscultation folder/psb-mastering-cardiac-auscultation/assets/common/assets/css/jplayer/pbar-ani.gif]

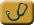

Supplement: Supplementary file 1 — A. PSB Mastering Cardiac Auscultation folder [file mep-13-10577-s001.zip › A. PSB Mastering Cardiac Auscultation folder/psb-mastering-cardiac-auscultation/assets/common/assets/images/button_stethoscope.gif]

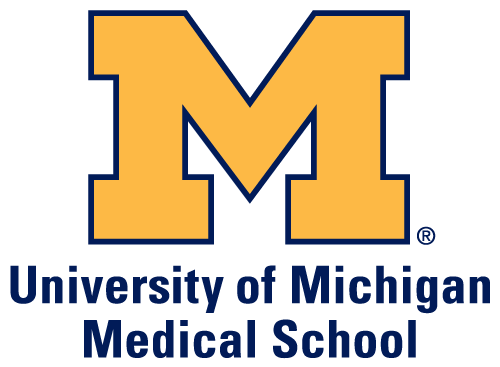

Supplement: Supplementary file 1 — A. PSB Mastering Cardiac Auscultation folder [file mep-13-10577-s001.zip › A. PSB Mastering Cardiac Auscultation folder/psb-mastering-cardiac-auscultation/assets/common/assets/images/dashboard/mlogo]

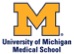

Supplement: Supplementary file 1 — A. PSB Mastering Cardiac Auscultation folder [file mep-13-10577-s001.zip › A. PSB Mastering Cardiac Auscultation folder/psb-mastering-cardiac-auscultation/assets/common/assets/images/dashboard/mlogo.jpg]

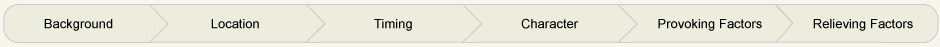

Supplement: Supplementary file 1 — A. PSB Mastering Cardiac Auscultation folder [file mep-13-10577-s001.zip › A. PSB Mastering Cardiac Auscultation folder/psb-mastering-cardiac-auscultation/assets/common/assets/images/roadmap/angina_navbar_1.png]

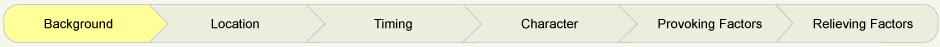

Supplement: Supplementary file 1 — A. PSB Mastering Cardiac Auscultation folder [file mep-13-10577-s001.zip › A. PSB Mastering Cardiac Auscultation folder/psb-mastering-cardiac-auscultation/assets/common/assets/images/roadmap/angina_navbar_2.png]

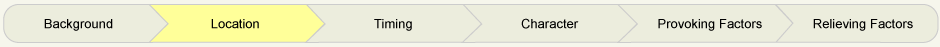

Supplement: Supplementary file 1 — A. PSB Mastering Cardiac Auscultation folder [file mep-13-10577-s001.zip › A. PSB Mastering Cardiac Auscultation folder/psb-mastering-cardiac-auscultation/assets/common/assets/images/roadmap/angina_navbar_3.png]

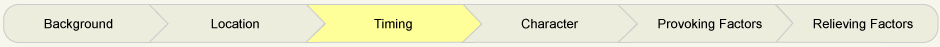

Supplement: Supplementary file 1 — A. PSB Mastering Cardiac Auscultation folder [file mep-13-10577-s001.zip › A. PSB Mastering Cardiac Auscultation folder/psb-mastering-cardiac-auscultation/assets/common/assets/images/roadmap/angina_navbar_4.png]

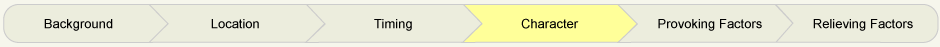

Supplement: Supplementary file 1 — A. PSB Mastering Cardiac Auscultation folder [file mep-13-10577-s001.zip › A. PSB Mastering Cardiac Auscultation folder/psb-mastering-cardiac-auscultation/assets/common/assets/images/roadmap/angina_navbar_5.png]

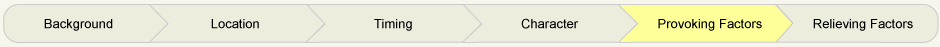

Supplement: Supplementary file 1 — A. PSB Mastering Cardiac Auscultation folder [file mep-13-10577-s001.zip › A. PSB Mastering Cardiac Auscultation folder/psb-mastering-cardiac-auscultation/assets/common/assets/images/roadmap/angina_navbar_6.png]

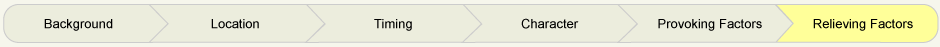

Supplement: Supplementary file 1 — A. PSB Mastering Cardiac Auscultation folder [file mep-13-10577-s001.zip › A. PSB Mastering Cardiac Auscultation folder/psb-mastering-cardiac-auscultation/assets/common/assets/images/roadmap/angina_navbar_7.png]

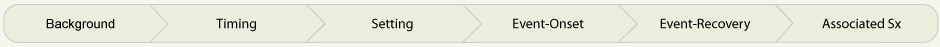

Supplement: Supplementary file 1 — A. PSB Mastering Cardiac Auscultation folder [file mep-13-10577-s001.zip › A. PSB Mastering Cardiac Auscultation folder/psb-mastering-cardiac-auscultation/assets/common/assets/images/roadmap/palp_navbar_1.png]

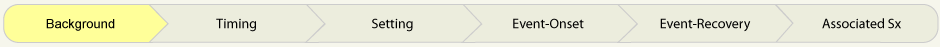

Supplement: Supplementary file 1 — A. PSB Mastering Cardiac Auscultation folder [file mep-13-10577-s001.zip › A. PSB Mastering Cardiac Auscultation folder/psb-mastering-cardiac-auscultation/assets/common/assets/images/roadmap/palp_navbar_2.png]

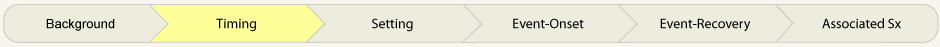

Supplement: Supplementary file 1 — A. PSB Mastering Cardiac Auscultation folder [file mep-13-10577-s001.zip › A. PSB Mastering Cardiac Auscultation folder/psb-mastering-cardiac-auscultation/assets/common/assets/images/roadmap/palp_navbar_3.png]

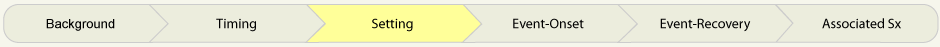

Supplement: Supplementary file 1 — A. PSB Mastering Cardiac Auscultation folder [file mep-13-10577-s001.zip › A. PSB Mastering Cardiac Auscultation folder/psb-mastering-cardiac-auscultation/assets/common/assets/images/roadmap/palp_navbar_4.png]

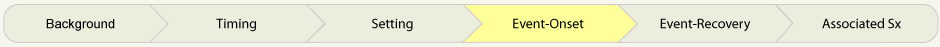

Supplement: Supplementary file 1 — A. PSB Mastering Cardiac Auscultation folder [file mep-13-10577-s001.zip › A. PSB Mastering Cardiac Auscultation folder/psb-mastering-cardiac-auscultation/assets/common/assets/images/roadmap/palp_navbar_5.png]

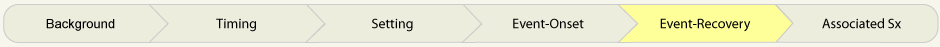

Supplement: Supplementary file 1 — A. PSB Mastering Cardiac Auscultation folder [file mep-13-10577-s001.zip › A. PSB Mastering Cardiac Auscultation folder/psb-mastering-cardiac-auscultation/assets/common/assets/images/roadmap/palp_navbar_6.png]

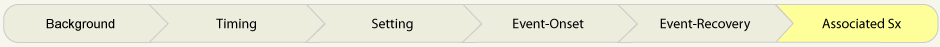

Supplement: Supplementary file 1 — A. PSB Mastering Cardiac Auscultation folder [file mep-13-10577-s001.zip › A. PSB Mastering Cardiac Auscultation folder/psb-mastering-cardiac-auscultation/assets/common/assets/images/roadmap/palp_navbar_7.png]

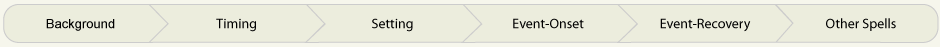

Supplement: Supplementary file 1 — A. PSB Mastering Cardiac Auscultation folder [file mep-13-10577-s001.zip › A. PSB Mastering Cardiac Auscultation folder/psb-mastering-cardiac-auscultation/assets/common/assets/images/roadmap/syncope_navbar_1.png]

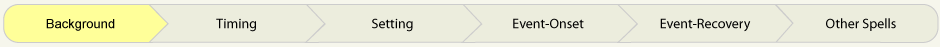

Supplement: Supplementary file 1 — A. PSB Mastering Cardiac Auscultation folder [file mep-13-10577-s001.zip › A. PSB Mastering Cardiac Auscultation folder/psb-mastering-cardiac-auscultation/assets/common/assets/images/roadmap/syncope_navbar_2.png]

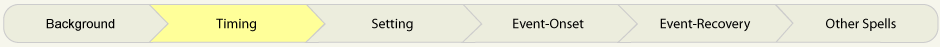

Supplement: Supplementary file 1 — A. PSB Mastering Cardiac Auscultation folder [file mep-13-10577-s001.zip › A. PSB Mastering Cardiac Auscultation folder/psb-mastering-cardiac-auscultation/assets/common/assets/images/roadmap/syncope_navbar_3.png]

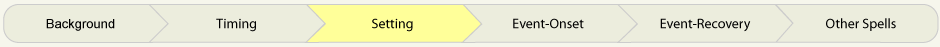

Supplement: Supplementary file 1 — A. PSB Mastering Cardiac Auscultation folder [file mep-13-10577-s001.zip › A. PSB Mastering Cardiac Auscultation folder/psb-mastering-cardiac-auscultation/assets/common/assets/images/roadmap/syncope_navbar_4.png]

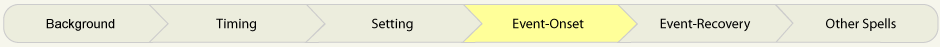

Supplement: Supplementary file 1 — A. PSB Mastering Cardiac Auscultation folder [file mep-13-10577-s001.zip › A. PSB Mastering Cardiac Auscultation folder/psb-mastering-cardiac-auscultation/assets/common/assets/images/roadmap/syncope_navbar_5.png]

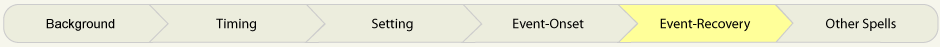

Supplement: Supplementary file 1 — A. PSB Mastering Cardiac Auscultation folder [file mep-13-10577-s001.zip › A. PSB Mastering Cardiac Auscultation folder/psb-mastering-cardiac-auscultation/assets/common/assets/images/roadmap/syncope_navbar_6.png]

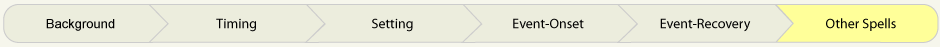

Supplement: Supplementary file 1 — A. PSB Mastering Cardiac Auscultation folder [file mep-13-10577-s001.zip › A. PSB Mastering Cardiac Auscultation folder/psb-mastering-cardiac-auscultation/assets/common/assets/images/roadmap/syncope_navbar_7.png]

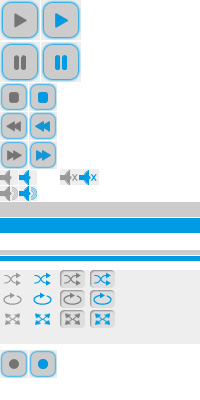

Supplement: Supplementary file 1 — A. PSB Mastering Cardiac Auscultation folder [file mep-13-10577-s001.zip › A. PSB Mastering Cardiac Auscultation folder/psb-mastering-cardiac-auscultation/assets/common_v2012/assets/css/blue.monday/jplayer.blue.monday.jpg]

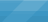

Supplement: Supplementary file 1 — A. PSB Mastering Cardiac Auscultation folder [file mep-13-10577-s001.zip › A. PSB Mastering Cardiac Auscultation folder/psb-mastering-cardiac-auscultation/assets/common_v2012/assets/css/blue.monday/jplayer.blue.monday.seeking.gif]

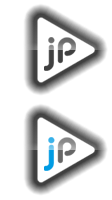

Supplement: Supplementary file 1 — A. PSB Mastering Cardiac Auscultation folder [file mep-13-10577-s001.zip › A. PSB Mastering Cardiac Auscultation folder/psb-mastering-cardiac-auscultation/assets/common_v2012/assets/css/blue.monday/jplayer.blue.monday.video.play.png]

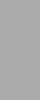

Supplement: Supplementary file 1 — A. PSB Mastering Cardiac Auscultation folder [file mep-13-10577-s001.zip › A. PSB Mastering Cardiac Auscultation folder/psb-mastering-cardiac-auscultation/assets/common_v2012/assets/css/images/ui-bg_flat_0_aaaaaa_40x100.png]

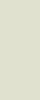

Supplement: Supplementary file 1 — A. PSB Mastering Cardiac Auscultation folder [file mep-13-10577-s001.zip › A. PSB Mastering Cardiac Auscultation folder/psb-mastering-cardiac-auscultation/assets/common_v2012/assets/css/images/ui-bg_flat_75_e1e1cf_40x100.png]

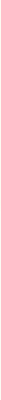

Supplement: Supplementary file 1 — A. PSB Mastering Cardiac Auscultation folder [file mep-13-10577-s001.zip › A. PSB Mastering Cardiac Auscultation folder/psb-mastering-cardiac-auscultation/assets/common_v2012/assets/css/images/ui-bg_glass_55_fbf9ee_1x400.png]

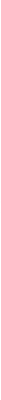

Supplement: Supplementary file 1 — A. PSB Mastering Cardiac Auscultation folder [file mep-13-10577-s001.zip › A. PSB Mastering Cardiac Auscultation folder/psb-mastering-cardiac-auscultation/assets/common_v2012/assets/css/images/ui-bg_glass_65_ffffff_1x400.png]

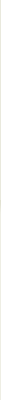

Supplement: Supplementary file 1 — A. PSB Mastering Cardiac Auscultation folder [file mep-13-10577-s001.zip › A. PSB Mastering Cardiac Auscultation folder/psb-mastering-cardiac-auscultation/assets/common_v2012/assets/css/images/ui-bg_glass_75_e1e1cf_1x400.png]

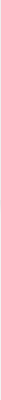

Supplement: Supplementary file 1 — A. PSB Mastering Cardiac Auscultation folder [file mep-13-10577-s001.zip › A. PSB Mastering Cardiac Auscultation folder/psb-mastering-cardiac-auscultation/assets/common_v2012/assets/css/images/ui-bg_glass_75_e6e6e6_1x400.png]

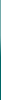

Supplement: Supplementary file 1 — A. PSB Mastering Cardiac Auscultation folder [file mep-13-10577-s001.zip › A. PSB Mastering Cardiac Auscultation folder/psb-mastering-cardiac-auscultation/assets/common_v2012/assets/css/images/ui-bg_highlight-soft_75_006666_1x100.png]

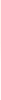

Supplement: Supplementary file 1 — A. PSB Mastering Cardiac Auscultation folder [file mep-13-10577-s001.zip › A. PSB Mastering Cardiac Auscultation folder/psb-mastering-cardiac-auscultation/assets/common_v2012/assets/css/images/ui-bg_inset-soft_95_fef1ec_1x100.png]

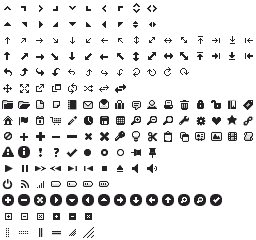

Supplement: Supplementary file 1 — A. PSB Mastering Cardiac Auscultation folder [file mep-13-10577-s001.zip › A. PSB Mastering Cardiac Auscultation folder/psb-mastering-cardiac-auscultation/assets/common_v2012/assets/css/images/ui-icons_222222_256x240.png]

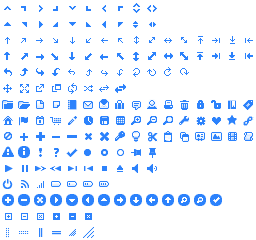

Supplement: Supplementary file 1 — A. PSB Mastering Cardiac Auscultation folder [file mep-13-10577-s001.zip › A. PSB Mastering Cardiac Auscultation folder/psb-mastering-cardiac-auscultation/assets/common_v2012/assets/css/images/ui-icons_2e83ff_256x240.png]

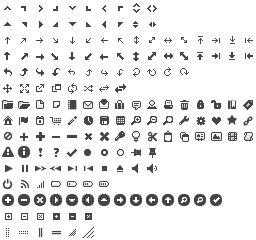

Supplement: Supplementary file 1 — A. PSB Mastering Cardiac Auscultation folder [file mep-13-10577-s001.zip › A. PSB Mastering Cardiac Auscultation folder/psb-mastering-cardiac-auscultation/assets/common_v2012/assets/css/images/ui-icons_454545_256x240.png]

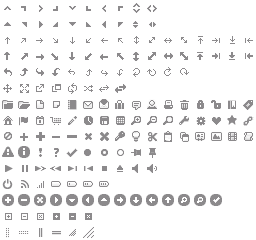

Supplement: Supplementary file 1 — A. PSB Mastering Cardiac Auscultation folder [file mep-13-10577-s001.zip › A. PSB Mastering Cardiac Auscultation folder/psb-mastering-cardiac-auscultation/assets/common_v2012/assets/css/images/ui-icons_888888_256x240.png]

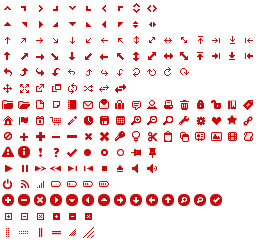

Supplement: Supplementary file 1 — A. PSB Mastering Cardiac Auscultation folder [file mep-13-10577-s001.zip › A. PSB Mastering Cardiac Auscultation folder/psb-mastering-cardiac-auscultation/assets/common_v2012/assets/css/images/ui-icons_cd0a0a_256x240.png]

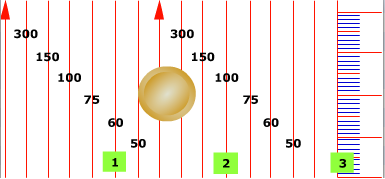

Supplement: Supplementary file 1 — A. PSB Mastering Cardiac Auscultation folder [file mep-13-10577-s001.zip › A. PSB Mastering Cardiac Auscultation folder/psb-mastering-cardiac-auscultation/assets/common_v2012/assets/images/ecg/scale.png]

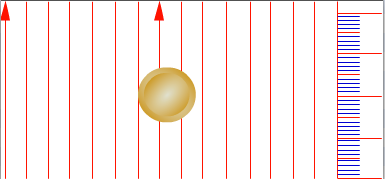

Supplement: Supplementary file 1 — A. PSB Mastering Cardiac Auscultation folder [file mep-13-10577-s001.zip › A. PSB Mastering Cardiac Auscultation folder/psb-mastering-cardiac-auscultation/assets/common_v2012/assets/images/ecg/scale_wo_numbers.png]

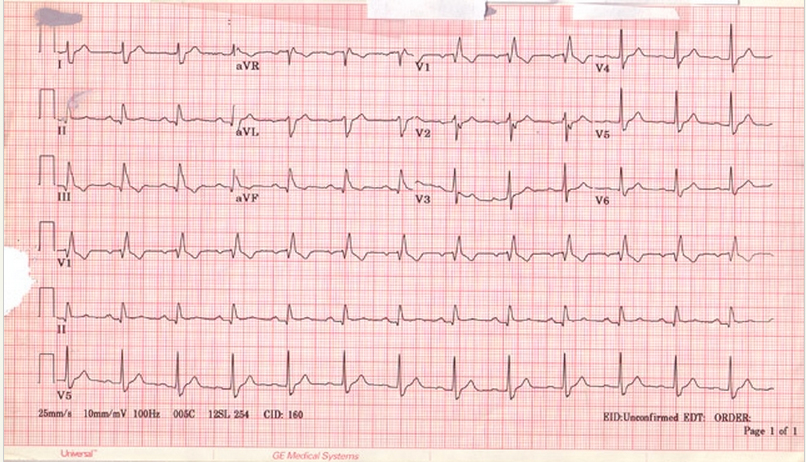

Supplement: Supplementary file 1 — A. PSB Mastering Cardiac Auscultation folder [file mep-13-10577-s001.zip › A. PSB Mastering Cardiac Auscultation folder/psb-mastering-cardiac-auscultation/assets/common_v2012/assets/images/ecg/set01case01.jpg]

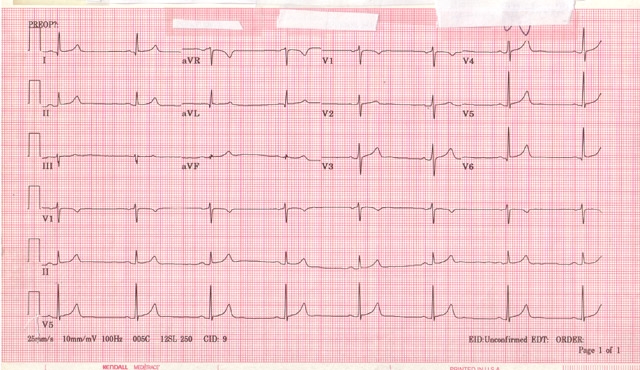

Supplement: Supplementary file 1 — A. PSB Mastering Cardiac Auscultation folder [file mep-13-10577-s001.zip › A. PSB Mastering Cardiac Auscultation folder/psb-mastering-cardiac-auscultation/assets/common_v2012/assets/images/ecg/set01case02.jpg]

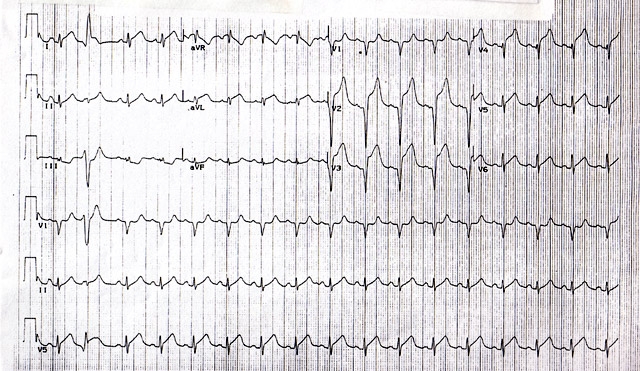

Supplement: Supplementary file 1 — A. PSB Mastering Cardiac Auscultation folder [file mep-13-10577-s001.zip › A. PSB Mastering Cardiac Auscultation folder/psb-mastering-cardiac-auscultation/assets/common_v2012/assets/images/ecg/set01case03.jpg]

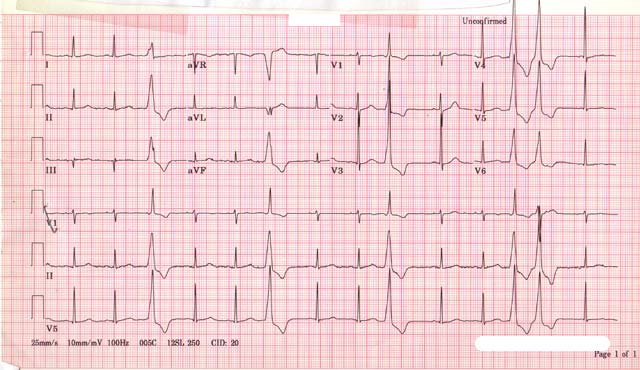

Supplement: Supplementary file 1 — A. PSB Mastering Cardiac Auscultation folder [file mep-13-10577-s001.zip › A. PSB Mastering Cardiac Auscultation folder/psb-mastering-cardiac-auscultation/assets/common_v2012/assets/images/ecg/set01case04.jpg]

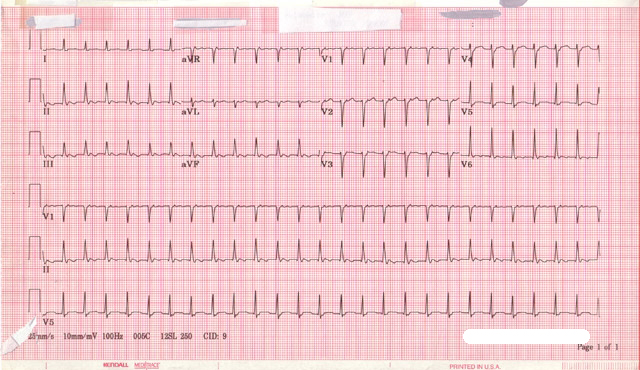

Supplement: Supplementary file 1 — A. PSB Mastering Cardiac Auscultation folder [file mep-13-10577-s001.zip › A. PSB Mastering Cardiac Auscultation folder/psb-mastering-cardiac-auscultation/assets/common_v2012/assets/images/ecg/set01case05.jpg]

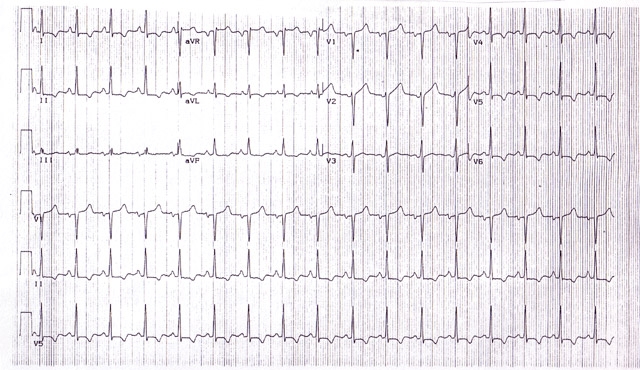

Supplement: Supplementary file 1 — A. PSB Mastering Cardiac Auscultation folder [file mep-13-10577-s001.zip › A. PSB Mastering Cardiac Auscultation folder/psb-mastering-cardiac-auscultation/assets/common_v2012/assets/images/ecg/set01case06.jpg]

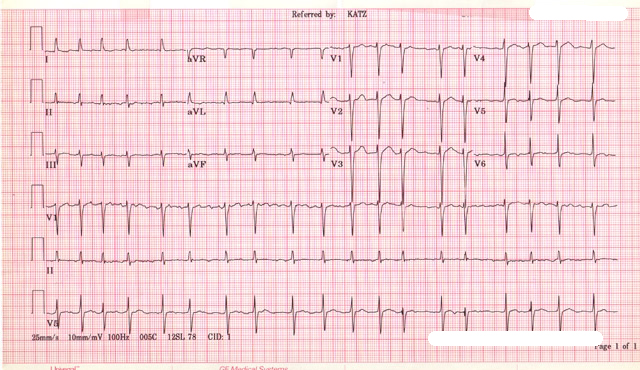

Supplement: Supplementary file 1 — A. PSB Mastering Cardiac Auscultation folder [file mep-13-10577-s001.zip › A. PSB Mastering Cardiac Auscultation folder/psb-mastering-cardiac-auscultation/assets/common_v2012/assets/images/ecg/set01case07.jpg]

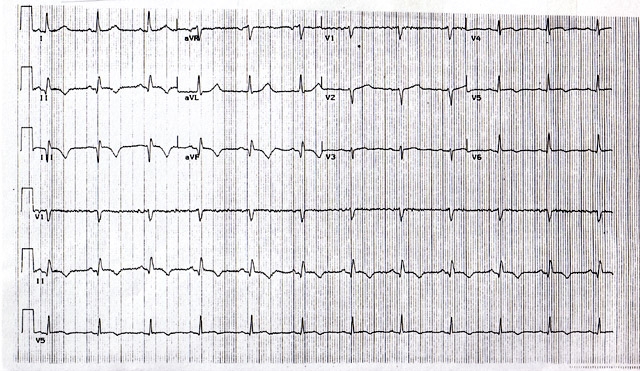

Supplement: Supplementary file 1 — A. PSB Mastering Cardiac Auscultation folder [file mep-13-10577-s001.zip › A. PSB Mastering Cardiac Auscultation folder/psb-mastering-cardiac-auscultation/assets/common_v2012/assets/images/ecg/set01case08.jpg]

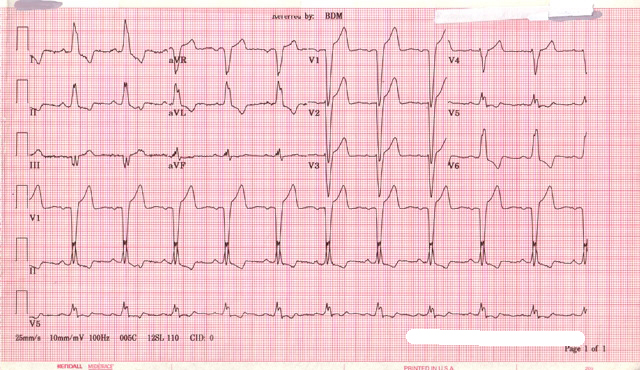

Supplement: Supplementary file 1 — A. PSB Mastering Cardiac Auscultation folder [file mep-13-10577-s001.zip › A. PSB Mastering Cardiac Auscultation folder/psb-mastering-cardiac-auscultation/assets/common_v2012/assets/images/ecg/set01case09.jpg]

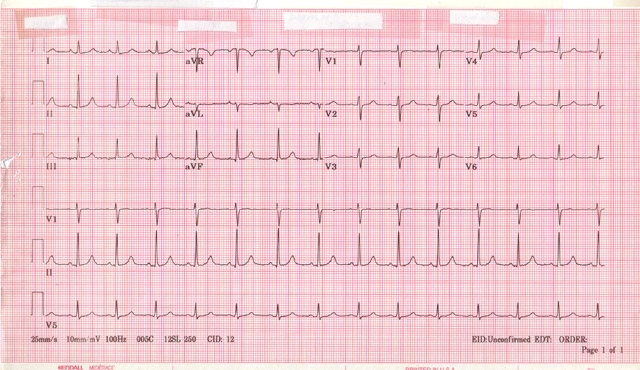

Supplement: Supplementary file 1 — A. PSB Mastering Cardiac Auscultation folder [file mep-13-10577-s001.zip › A. PSB Mastering Cardiac Auscultation folder/psb-mastering-cardiac-auscultation/assets/common_v2012/assets/images/ecg/set01case10.jpg]

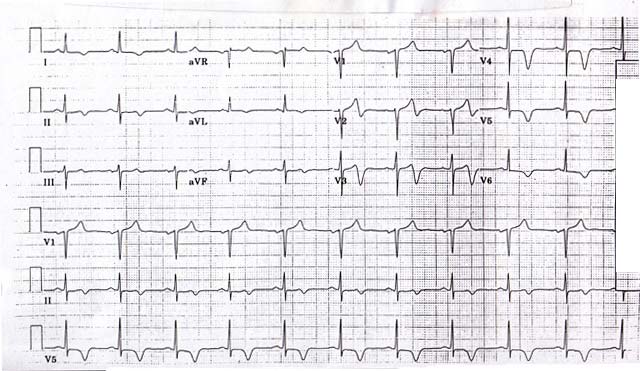

Supplement: Supplementary file 1 — A. PSB Mastering Cardiac Auscultation folder [file mep-13-10577-s001.zip › A. PSB Mastering Cardiac Auscultation folder/psb-mastering-cardiac-auscultation/assets/common_v2012/assets/images/ecg/set02case01.jpg]

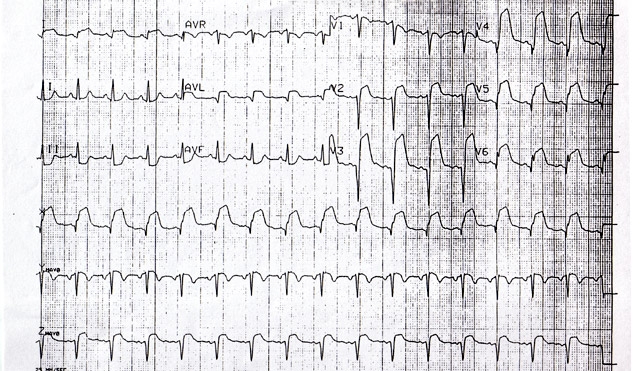

Supplement: Supplementary file 1 — A. PSB Mastering Cardiac Auscultation folder [file mep-13-10577-s001.zip › A. PSB Mastering Cardiac Auscultation folder/psb-mastering-cardiac-auscultation/assets/common_v2012/assets/images/ecg/set02case02.jpg]

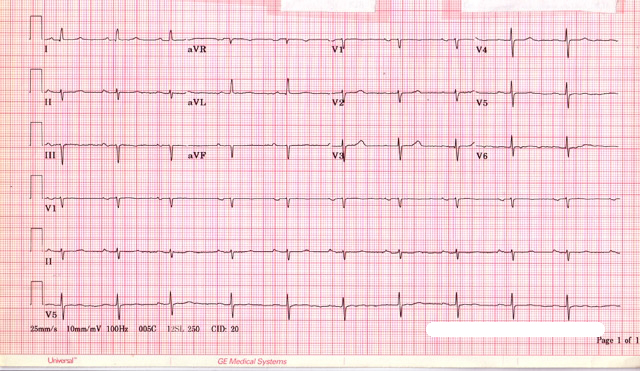

Supplement: Supplementary file 1 — A. PSB Mastering Cardiac Auscultation folder [file mep-13-10577-s001.zip › A. PSB Mastering Cardiac Auscultation folder/psb-mastering-cardiac-auscultation/assets/common_v2012/assets/images/ecg/set02case03.jpg]

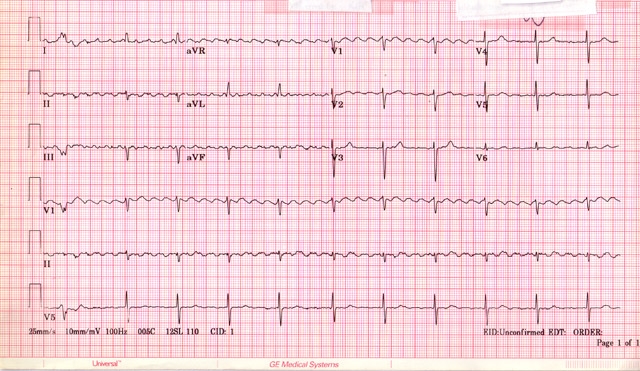

Supplement: Supplementary file 1 — A. PSB Mastering Cardiac Auscultation folder [file mep-13-10577-s001.zip › A. PSB Mastering Cardiac Auscultation folder/psb-mastering-cardiac-auscultation/assets/common_v2012/assets/images/ecg/set02case04.jpg]

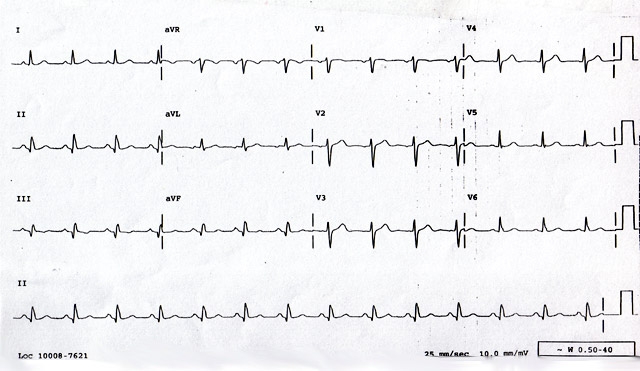

Supplement: Supplementary file 1 — A. PSB Mastering Cardiac Auscultation folder [file mep-13-10577-s001.zip › A. PSB Mastering Cardiac Auscultation folder/psb-mastering-cardiac-auscultation/assets/common_v2012/assets/images/ecg/set02case05.jpg]

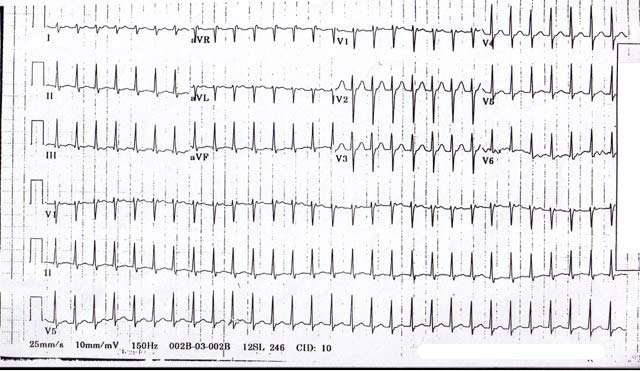

Supplement: Supplementary file 1 — A. PSB Mastering Cardiac Auscultation folder [file mep-13-10577-s001.zip › A. PSB Mastering Cardiac Auscultation folder/psb-mastering-cardiac-auscultation/assets/common_v2012/assets/images/ecg/set02case06.jpg]

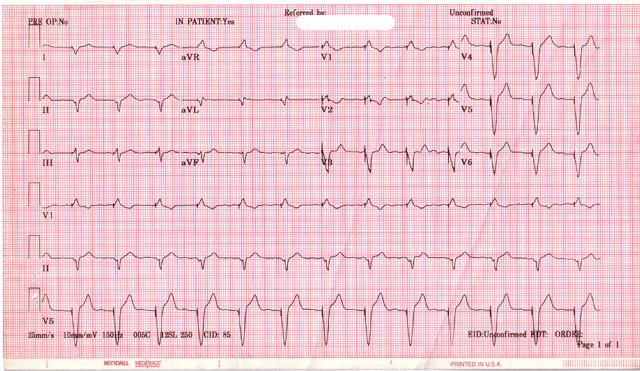

Supplement: Supplementary file 1 — A. PSB Mastering Cardiac Auscultation folder [file mep-13-10577-s001.zip › A. PSB Mastering Cardiac Auscultation folder/psb-mastering-cardiac-auscultation/assets/common_v2012/assets/images/ecg/set02case07.jpg]

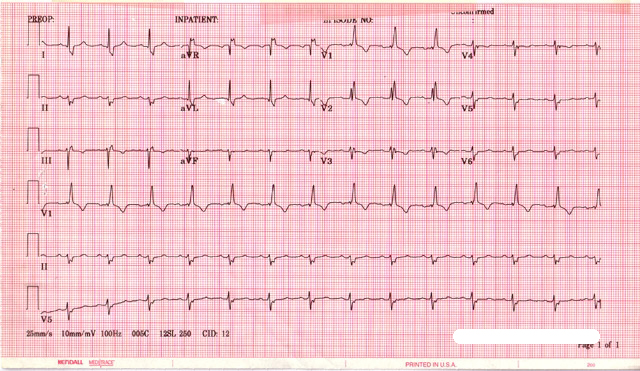

Supplement: Supplementary file 1 — A. PSB Mastering Cardiac Auscultation folder [file mep-13-10577-s001.zip › A. PSB Mastering Cardiac Auscultation folder/psb-mastering-cardiac-auscultation/assets/common_v2012/assets/images/ecg/set02case08.jpg]

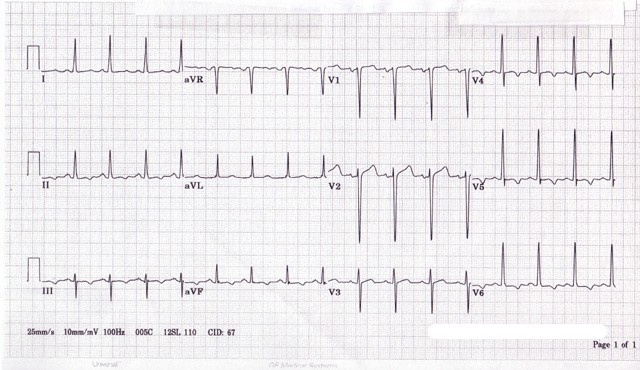

Supplement: Supplementary file 1 — A. PSB Mastering Cardiac Auscultation folder [file mep-13-10577-s001.zip › A. PSB Mastering Cardiac Auscultation folder/psb-mastering-cardiac-auscultation/assets/common_v2012/assets/images/ecg/set02case09.jpg]

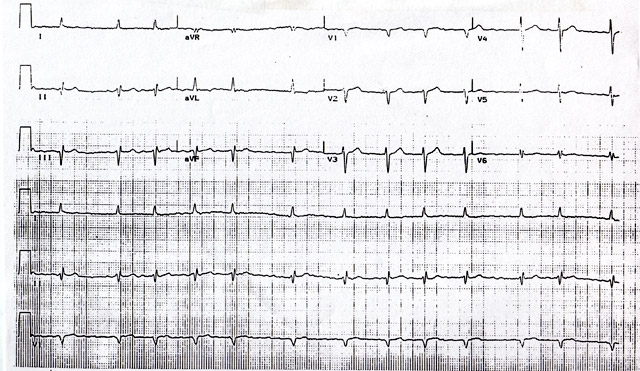

Supplement: Supplementary file 1 — A. PSB Mastering Cardiac Auscultation folder [file mep-13-10577-s001.zip › A. PSB Mastering Cardiac Auscultation folder/psb-mastering-cardiac-auscultation/assets/common_v2012/assets/images/ecg/set02case10.jpg]

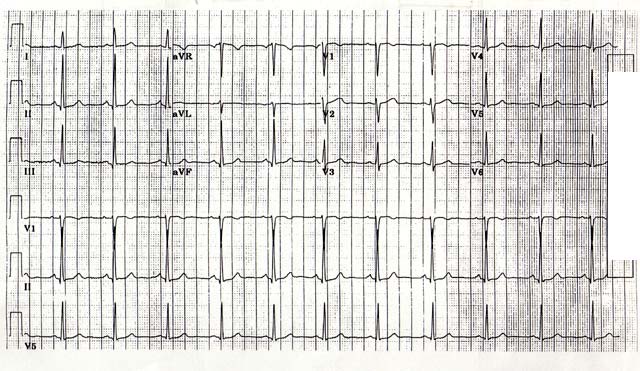

Supplement: Supplementary file 1 — A. PSB Mastering Cardiac Auscultation folder [file mep-13-10577-s001.zip › A. PSB Mastering Cardiac Auscultation folder/psb-mastering-cardiac-auscultation/assets/common_v2012/assets/images/ecg/set03case01.jpg]

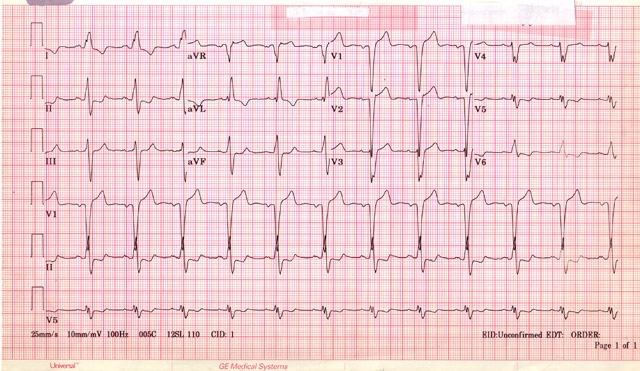

Supplement: Supplementary file 1 — A. PSB Mastering Cardiac Auscultation folder [file mep-13-10577-s001.zip › A. PSB Mastering Cardiac Auscultation folder/psb-mastering-cardiac-auscultation/assets/common_v2012/assets/images/ecg/set03case02.jpg]

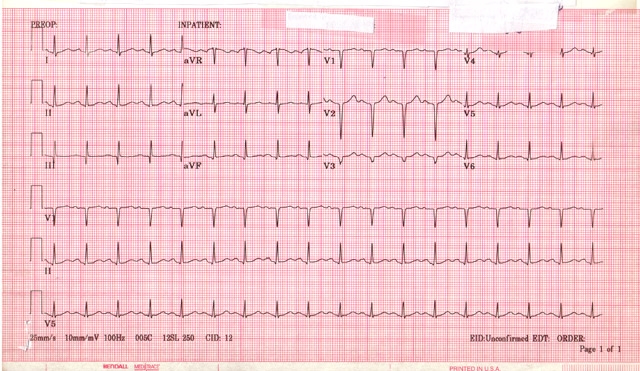

Supplement: Supplementary file 1 — A. PSB Mastering Cardiac Auscultation folder [file mep-13-10577-s001.zip › A. PSB Mastering Cardiac Auscultation folder/psb-mastering-cardiac-auscultation/assets/common_v2012/assets/images/ecg/set03case03.jpg]

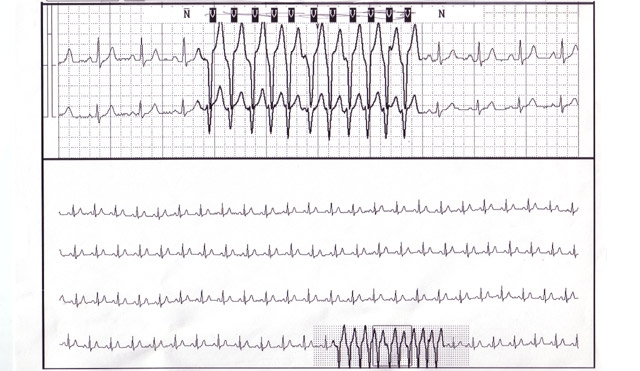

Supplement: Supplementary file 1 — A. PSB Mastering Cardiac Auscultation folder [file mep-13-10577-s001.zip › A. PSB Mastering Cardiac Auscultation folder/psb-mastering-cardiac-auscultation/assets/common_v2012/assets/images/ecg/set03case04.jpg]

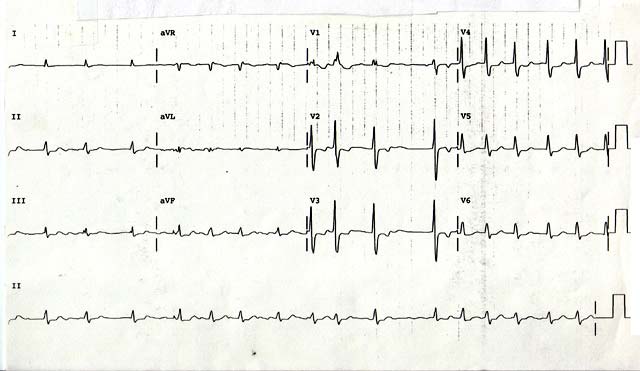

Supplement: Supplementary file 1 — A. PSB Mastering Cardiac Auscultation folder [file mep-13-10577-s001.zip › A. PSB Mastering Cardiac Auscultation folder/psb-mastering-cardiac-auscultation/assets/common_v2012/assets/images/ecg/set03case05.jpg]

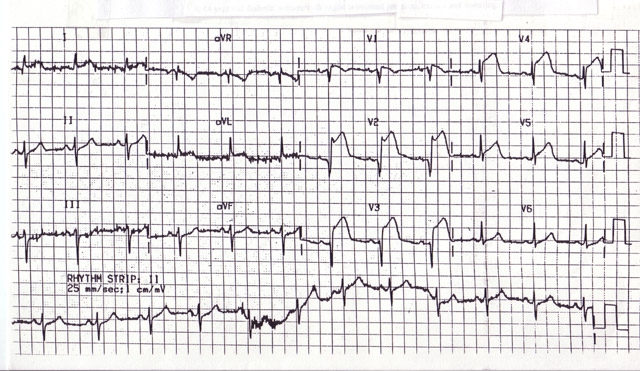

Supplement: Supplementary file 1 — A. PSB Mastering Cardiac Auscultation folder [file mep-13-10577-s001.zip › A. PSB Mastering Cardiac Auscultation folder/psb-mastering-cardiac-auscultation/assets/common_v2012/assets/images/ecg/set03case06.jpg]

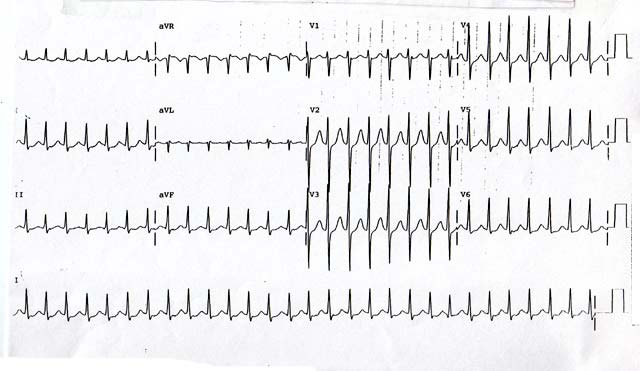

Supplement: Supplementary file 1 — A. PSB Mastering Cardiac Auscultation folder [file mep-13-10577-s001.zip › A. PSB Mastering Cardiac Auscultation folder/psb-mastering-cardiac-auscultation/assets/common_v2012/assets/images/ecg/set03case07.jpg]

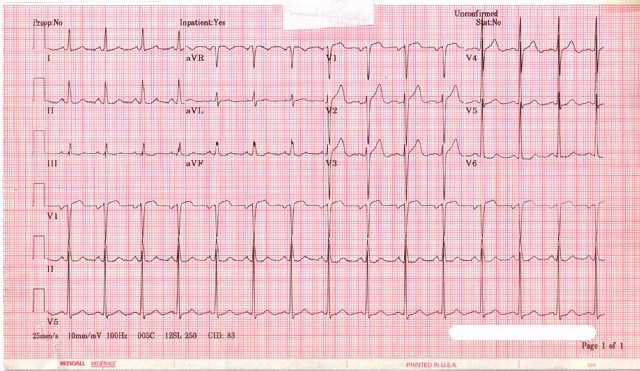

Supplement: Supplementary file 1 — A. PSB Mastering Cardiac Auscultation folder [file mep-13-10577-s001.zip › A. PSB Mastering Cardiac Auscultation folder/psb-mastering-cardiac-auscultation/assets/common_v2012/assets/images/ecg/set03case08.jpg]

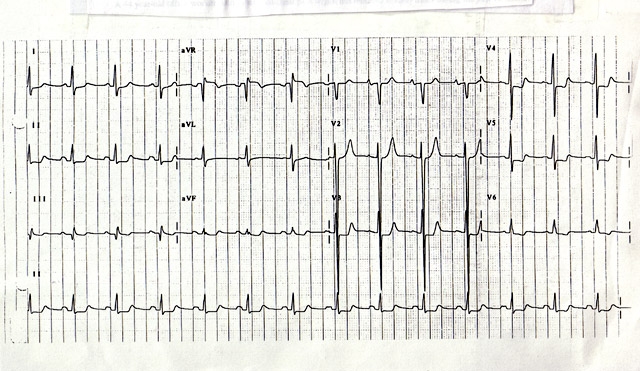

Supplement: Supplementary file 1 — A. PSB Mastering Cardiac Auscultation folder [file mep-13-10577-s001.zip › A. PSB Mastering Cardiac Auscultation folder/psb-mastering-cardiac-auscultation/assets/common_v2012/assets/images/ecg/set03case09.jpg]
