# Supplementary material for: Professional Skill Builder: Mastering Cardiac Auscultation in Under 4 Hours
Source: MedEdPORTAL. 2017 May 8;13:10577. doi: 10.15766/mep_2374-8265.10577 (PMC6338255; doi:10.15766/mep_2374-8265.10577)
Supplement: Supplementary file 1 — A. PSB Mastering Cardiac Auscultation folder [file mep-13-10577-s001.zip › A. PSB Mastering Cardiac Auscultation folder/psb-mastering-cardiac-auscultation/assets/repo/cards_mini_case_01/c_01_1.html]

Professional Skill Builder | Cardiology Case 1


Cardiology Case 1

:   Next
:   Previous
:   (screen 1 of 6)

**Instructions:**  After reading the patient summary, click
on the stethoscope head to listen to the heart sound. Then click on the
diagram that best matches what you hear.

A 64-year-old business man is seen because of the recent onset of exertional chest discomfort which is classic for angina pectoris. No other symptoms. **PMH.** Negative. **FH.** Positive for CAD (brother has had CAB). SH. Discontinued cigarette smoking 5 years ago (40 pack-years). **Physical Examination**: BP 126/70, P 70, R 12, Ht. 5'11", Wt. 171. General physical examination normal for age. Carotid and jugular pulses normal. No cardiac enlargement. Apex impulse normal. The following was heard on auscultation.

APEX, BELL (Supine)

:   **CORRECT**

    This is trochaic metre.

    Play the phono and then, if you wish, click on the incorrect diagrams
    to compare heart sounds.

    Move on.
:   **INCORRECT**

    S_2_ is not louder than S_1_. The heart sound matching this diagram is playing now. Please try again.
:   **INCORRECT**

    The patient does not have an S_4_. The heart sound matching this diagram is playing now. Please try again.

- Play
  Phono
