# Supplementary material for: Professional Skill Builder: Mastering Cardiac Auscultation in Under 4 Hours
Source: MedEdPORTAL. 2017 May 8;13:10577. doi: 10.15766/mep_2374-8265.10577 (PMC6338255; doi:10.15766/mep_2374-8265.10577)
Supplement: Supplementary file 1 — A. PSB Mastering Cardiac Auscultation folder [file mep-13-10577-s001.zip › A. PSB Mastering Cardiac Auscultation folder/psb-mastering-cardiac-auscultation/assets/repo/cards_mini_case_01/c_01_3.html]

Professional Skill Builder | Cardiology Case 1


Cardiology Case 1

:   Next
:   Previous
:   (screen 3 of 6)

**Instructions:**  Identify the following heart sound.

In this location the following was heard:

AORTIC Area, DIAPHRAGM (Supine)

:   **INCORRECT**

    There is no S_3_  
    The heart sound matching this diagram is playing now. Please try again.
:   **CORRECT**

    This is an iambic metre.

    Play the phono and then, if you wish, click on the incorrect diagrams
    to compare heart sounds.

    Move on.
:   **INCORRECT**

    S_2_ is not split.  
    The heart sound matching this diagram is playing now. Select again.

- Play
  Phono
