# Supplementary material for: Professional Skill Builder: Mastering Cardiac Auscultation in Under 4 Hours
Source: MedEdPORTAL. 2017 May 8;13:10577. doi: 10.15766/mep_2374-8265.10577 (PMC6338255; doi:10.15766/mep_2374-8265.10577)
Supplement: Supplementary file 1 — A. PSB Mastering Cardiac Auscultation folder [file mep-13-10577-s001.zip › A. PSB Mastering Cardiac Auscultation folder/psb-mastering-cardiac-auscultation/assets/repo/cards_mini_case_01/c_01_5.html]

Professional Skill Builder | Cardiology Case 1


Cardiology Case 1

:   Next
:   Previous
:   (screen 5 of 6)

In fact, this patient's clinical examination was entirely normal. However,
he did have a standard treadmill exercise test.

#### Baseline interpretation:

Flat T waves in I and V6. Otherwise within normal limits.

#### Post exercise ECG interpretation:

Marked, down-sloping ST depression. Suggests significant myocardial ischemia.
