# Supplementary material for: Professional Skill Builder: Mastering Cardiac Auscultation in Under 4 Hours
Source: MedEdPORTAL. 2017 May 8;13:10577. doi: 10.15766/mep_2374-8265.10577 (PMC6338255; doi:10.15766/mep_2374-8265.10577)
Supplement: Supplementary file 1 — A. PSB Mastering Cardiac Auscultation folder [file mep-13-10577-s001.zip › A. PSB Mastering Cardiac Auscultation folder/psb-mastering-cardiac-auscultation/assets/repo/cards_mini_case_01/c_01_6.html]

Professional Skill Builder | Cardiology Case 1


Cardiology Case 1

:   Next
:   Previous
:   (screen 6 of 6)

#### Summary:

*Click
to review auscultation.*

#### Lessons:

1. The main point of this case is that many patients with significant coronary disease have no abnormality on physical examination. However, cardiac auscultation is often helpful in patients with coronary artery disease as we shall demonstrate in later cases.
2. The fact that BP was normal and there was no S_3_, S_4_, murmur, or rhythm irregularity, however, does have significance. It is a relatively favorable sign.
3. The absence of abnormality on the examination makes it more likely that LV function is going to be relatively well preserved. This has prognostic value.
4. Fortunately for this patient, another stress test with imaging was performed. It demonstrated severe anteroseptal ischemia. This was followed by coronary angiography which demonstrated a high grade LAD lesion. He elected to have PTCA. It was successful. He is now pain-free.

#### *Case 1 is now finished.*
