# Supplementary material for: Professional Skill Builder: Mastering Cardiac Auscultation in Under 4 Hours
Source: MedEdPORTAL. 2017 May 8;13:10577. doi: 10.15766/mep_2374-8265.10577 (PMC6338255; doi:10.15766/mep_2374-8265.10577)
Supplement: Supplementary file 1 — A. PSB Mastering Cardiac Auscultation folder [file mep-13-10577-s001.zip › A. PSB Mastering Cardiac Auscultation folder/psb-mastering-cardiac-auscultation/assets/repo/cards_mini_case_02/c_02_1.html]

Professional Skill Builder | Cardiology Case 2


Cardiology Case 2

:   Next
:   Previous
:   (screen 1 of 5)

**Instructions:**  After reading the patient summary, click
on the stethoscope head to listen to the heart sound. Next, click on the
diagram that corresponds to what you hear.

A 55-year-old female administrator is seen because of chest pain which is classic for mild, stable, angina pectoris. She has never been hospitalized for any reason. **FH.** Strongly positive for CAD. **SH.** 1 pack per day cigarette smoker. **PMH.** Negative. **ROS.** Negative. **Physical Examination:** BP 120/72, P 74, R 12. Ht 5'5", Wt. 138. General examination entirely normal. No cardiac enlargement but the apex impulse is sustained with a palpable presystolic movement. The following was heard on auscultation.

APEX BELL, (Supine)

:   **INCORRECT**

    There is more than just a systolic murmur. The heart sound matching this diagram is playing now. Please try again.
:   **CORRECT**

    Play the phono and then, if you wish, click on the incorrect diagrams
    to compare heart sounds.

    Move on.
:   **INCORRECT**

    The patient does not have an S_3_. The heart sound matching this diagram is playing now. Please try again.

- Play
  Phono
