# Supplementary material for: Professional Skill Builder: Mastering Cardiac Auscultation in Under 4 Hours
Source: MedEdPORTAL. 2017 May 8;13:10577. doi: 10.15766/mep_2374-8265.10577 (PMC6338255; doi:10.15766/mep_2374-8265.10577)
Supplement: Supplementary file 1 — A. PSB Mastering Cardiac Auscultation folder [file mep-13-10577-s001.zip › A. PSB Mastering Cardiac Auscultation folder/psb-mastering-cardiac-auscultation/assets/repo/cards_mini_case_02/c_02_5.html]

Professional Skill Builder | Cardiology Case 2


Cardiology Case 2

:   Next
:   Previous
:   (screen 5 of 5)

#### Summary:

An echocardiogram confirmed the presence of an inferior myocardial infarct. There was also mild to moderate (1 to 2+) mitral regurgitation. The EF (Ejection Fraction) was 45%.

*Click
to review auscultation.*

#### Lessons:

1. This patient had had a silent inferior MI which was followed by the gradual onset of classic exertional angina.
2. The presence of an apical systolic murmur of mitral regurgitation is helpful and suggests LV dysfunction. This might affect your management, e.g., treatment with an ACE inhibitor.
3. Because ischemic myocardium is stiffer and less compliant than normal myocardium, patients with coronary artery disease often have an apical S_4_ which reflects impaired diastolic relaxation. In this case the forceful "atrial kick" was not only heard but also felt as a presystolic movement at the apex.
4. Silent MIs are surprisingly common. The Framingham Study showed a substantial number of citizens who turned up with diagnostic changes of MI on ECG and ECHO who denied ever having had chest pain or any other symptoms consistent with acute MI.

#### *Case 2 is now finished.*
