# Supplementary material for: Professional Skill Builder: Mastering Cardiac Auscultation in Under 4 Hours
Source: MedEdPORTAL. 2017 May 8;13:10577. doi: 10.15766/mep_2374-8265.10577 (PMC6338255; doi:10.15766/mep_2374-8265.10577)
Supplement: Supplementary file 1 — A. PSB Mastering Cardiac Auscultation folder [file mep-13-10577-s001.zip › A. PSB Mastering Cardiac Auscultation folder/psb-mastering-cardiac-auscultation/assets/repo/cards_mini_case_04/c_04_1.html]

Professional Skill Builder | Cardiology Case 4


Cardiology Case 4

:   Next
:   Previous
:   (screen 1 of 5)

**Instructions:**  After reading the patient summary, click
on the stethoscope head to listen to the heart sound. Then click on the
diagram that best matches what you hear.

A 39-year-old mathematics professor has a BP of 214/110 at the time of a periodic checkup. Previous BPs described as "borderline." No symptoms. **FH.** Negative. Very athletic. Runs 5-10 miles per week.   
**Physical examination:** Mild hypertensive retinopathy. Sustained double apex impule which is not displaced laterally. The following was heard on auscultation.

:   **INCORRECT**

    This is a mid-systolic click

    The heart sound matching this diagram is playing now. Please try again.
:   **INCORRECT**

    S_1_ is not a split. The heart sound matching this diagram is playing now. Please try again.
:   **CORRECT**

    This is a anapestic metre.

    Play the phono and then, if you wish, click on the incorrect diagrams
    to compare heart sounds.

    Move on.

- Play
  Phono
