# Supplementary material for: Professional Skill Builder: Mastering Cardiac Auscultation in Under 4 Hours
Source: MedEdPORTAL. 2017 May 8;13:10577. doi: 10.15766/mep_2374-8265.10577 (PMC6338255; doi:10.15766/mep_2374-8265.10577)
Supplement: Supplementary file 1 — A. PSB Mastering Cardiac Auscultation folder [file mep-13-10577-s001.zip › A. PSB Mastering Cardiac Auscultation folder/psb-mastering-cardiac-auscultation/assets/repo/cards_mini_case_04/c_04_2.html]

Professional Skill Builder | Cardiology Case 4


Cardiology Case 4

:   Next
:   Previous
:   (screen 2 of 5)

**Instructions:**  Review the patient's auscultation and
answer the questions below.

*Click
to review heart sound.*

##### This patient has:

- A

  Split S_1_
- B

  An S_4_
- C

  An S_4_ and an increased S_2_

:   **INCORRECT**

    Try again.
:   **INCORRECT**

    Yes, but there is more. Select again.
:   **CORRECT**

    Be sure to notice that S_2_ is markedly accentuated.

    Show Next Question

*Click
to review heart sound.*

##### The S_4_ in this patient is probably due to:

- A

  Early closure of the mitral valve
- B

  Very forceful left atrial contraction
- C

  Mitral stenosis

:   **INCORRECT**

    Try again.
:   **CORRECT**

    Move on to the next screen.
:   **INCORRECT**

    Select again.
