# Supplementary material for: Professional Skill Builder: Mastering Cardiac Auscultation in Under 4 Hours
Source: MedEdPORTAL. 2017 May 8;13:10577. doi: 10.15766/mep_2374-8265.10577 (PMC6338255; doi:10.15766/mep_2374-8265.10577)
Supplement: Supplementary file 1 — A. PSB Mastering Cardiac Auscultation folder [file mep-13-10577-s001.zip › A. PSB Mastering Cardiac Auscultation folder/psb-mastering-cardiac-auscultation/assets/repo/cards_mini_case_04/c_04_3.html]

Professional Skill Builder | Cardiology Case 4


Cardiology Case 4

:   Next
:   Previous
:   (screen 3 of 5)

**Instructions:**  Review the patient's auscultation and
answer the questions below.

*Click
to review heart sound.*

##### The forceful left atrial contraction is probably secondary to:

- A

  Congestive heart failure
- B

  Decreased left ventricular systolic pressure
- C

  Impaired diastolic relaxation due to left ventricular hypertrophy

:   **INCORRECT**

    Try again.
:   **INCORRECT**

    Select again.
:   **CORRECT**

    Show Next Question

*Click
to review heart sound.*

##### The loud S_2_ probably is due to:

- A

  Pulmonary valve closure
- B

  Elevated left atrial pressure
- C

  Forceful aortic valve closure secondary to his elevated diastolic blood pressure

:   **INCORRECT**

    Try again.
:   **INCORRECT**

    Select again.
:   **CORRECT**

    Move on to the next screen.
