# Supplementary material for: Professional Skill Builder: Mastering Cardiac Auscultation in Under 4 Hours
Source: MedEdPORTAL. 2017 May 8;13:10577. doi: 10.15766/mep_2374-8265.10577 (PMC6338255; doi:10.15766/mep_2374-8265.10577)
Supplement: Supplementary file 1 — A. PSB Mastering Cardiac Auscultation folder [file mep-13-10577-s001.zip › A. PSB Mastering Cardiac Auscultation folder/psb-mastering-cardiac-auscultation/assets/repo/cards_mini_case_04/c_04_4.html]

Professional Skill Builder | Cardiology Case 4


Cardiology Case 4

:   Next
:   Previous
:   (screen 4 of 5)

**Instructions:**  Differentiating an S4 from a normally
split S1 is often very difficult. Click on the buttons above for an explanation.
Begin with the BELL and then go to the DIAPHRAGM.

Click
to listen to Mentor

Click
to listen to Mentor
