# Supplementary material for: Professional Skill Builder: Mastering Cardiac Auscultation in Under 4 Hours
Source: MedEdPORTAL. 2017 May 8;13:10577. doi: 10.15766/mep_2374-8265.10577 (PMC6338255; doi:10.15766/mep_2374-8265.10577)
Supplement: Supplementary file 1 — A. PSB Mastering Cardiac Auscultation folder [file mep-13-10577-s001.zip › A. PSB Mastering Cardiac Auscultation folder/psb-mastering-cardiac-auscultation/assets/repo/cards_mini_case_04/c_04_5.html]

Professional Skill Builder | Cardiology Case 4


Cardiology Case 4

:   Next
:   Previous
:   (screen 5 of 5)

//

#### Summary:

This patient had a 2D echocardiogram which confirmed the presence of left
ventricular hypertrophy.

*Click
to review auscultation.*

#### Lessons:

1. Unlike the S_3_, the apical S_4_ is usually considered
   pathological and is often associated with an abnormal apex impulse.
2. It is often due to left ventricular hypertrophy, which is a common
   finding in patients with hypertension.
3. Hypertensive patients with significant left
   ventricular hypertrophy are at significantly higher
   risk for coronary artery disease (eg. myocardial infarction) than
   hypertensive patients with no evidence of left ventricular hypertrophy.
   Therefore this finding (the S_4_) could have a bearing on your
   management strategy, e.g., lipids, diet, exercise, etc.

**Note:**  
After age 75 many individuals have an audible S_4_ at the apex,
but these people will usually show LVH and impaired diastolic relaxation
on their echocardiogram. Though common in the elderly, an S_4_ is
not necessarily "normal."

#### *Case 4 is now finished.*
