# Supplementary material for: Professional Skill Builder: Mastering Cardiac Auscultation in Under 4 Hours
Source: MedEdPORTAL. 2017 May 8;13:10577. doi: 10.15766/mep_2374-8265.10577 (PMC6338255; doi:10.15766/mep_2374-8265.10577)
Supplement: Supplementary file 1 — A. PSB Mastering Cardiac Auscultation folder [file mep-13-10577-s001.zip › A. PSB Mastering Cardiac Auscultation folder/psb-mastering-cardiac-auscultation/assets/repo/cards_mini_case_06/c_06_1.html]

Professional Skill Builder | Cardiology Case 6


Cardiology Case 6

:   Next
:   Previous
:   (screen 1 of 4)

**Instructions:**  After reading the patient summary, click
on the stethoscope head to listen to the heart sound. Then click on the
diagram that best matches what you hear.

A 19-year-old female university sophomore is seen because of palpitation that comes on in the evening. Irregularity only--no fast pounding. No other symptoms. **FH.** Mother has heart murmur. **SH.** Drinks about10 cups of coffee/day. **Physical Examination:** BP 110/70, P 72, R 12, Ht. 5' 5", Wt. 130. Examination is negative except for two premature beats and the following:

APEX BELL, (Supine)

:   **CORRECT**

    Play the phono and then, if you wish, click on the incorrect diagrams
    to compare heart sounds.

    Move on.
:   **INCORRECT**

    The patient does not have splitting of S_1_. The heart sound matching this diagram is playing now. Please try again.
:   **INCORRECT**

    The patient does not have a S_3_. The heart sound matching this diagram is playing now. Please try again.

- Play
  Phono
