# Supplementary material for: Professional Skill Builder: Mastering Cardiac Auscultation in Under 4 Hours
Source: MedEdPORTAL. 2017 May 8;13:10577. doi: 10.15766/mep_2374-8265.10577 (PMC6338255; doi:10.15766/mep_2374-8265.10577)
Supplement: Supplementary file 1 — A. PSB Mastering Cardiac Auscultation folder [file mep-13-10577-s001.zip › A. PSB Mastering Cardiac Auscultation folder/psb-mastering-cardiac-auscultation/assets/repo/cards_mini_case_06/c_06_2.html]

Professional Skill Builder | Cardiology Case 6


Cardiology Case 6

:   Next
:   Previous
:   (screen 2 of 4)

**Instructions:**  Review the patient's auscultation and
answer the questions below.

*Click
to review heart sound.*

##### This patient has:

- A

  Mid-systolic click
- B

  A split S_1_
- C

  An S_3_

:   **CORRECT**

    Please move on.

    Show Next Question
:   **INCORRECT**

    Try again.
:   **INCORRECT**

    Try again.

*Click
to review heart sound.*

##### In a patient of this age, this finding at this location is:

- A

  Normal
- B

  Abnormal but insignificant; would not require follow-up.
- C

  Abnormal and significant; will require follow-up

:   **INCORRECT**

    It is not normal. Try again.
:   **INCORRECT**

    Select again.
:   **CORRECT**

    Abnormal and significant. It will requre INVESTIGATION and follow-up. Move on to the next screen.
