# Supplementary material for: Professional Skill Builder: Mastering Cardiac Auscultation in Under 4 Hours
Source: MedEdPORTAL. 2017 May 8;13:10577. doi: 10.15766/mep_2374-8265.10577 (PMC6338255; doi:10.15766/mep_2374-8265.10577)
Supplement: Supplementary file 1 — A. PSB Mastering Cardiac Auscultation folder [file mep-13-10577-s001.zip › A. PSB Mastering Cardiac Auscultation folder/psb-mastering-cardiac-auscultation/assets/repo/cards_mini_case_06/c_06_3.html]

Professional Skill Builder | Cardiology Case 6


Cardiology Case 6

:   Next
:   Previous
:   (screen 3 of 4)

**Instructions:**  Review the patient's auscultation and
answer the question below.

*Click
to review auscultation.*

##### It is probably caused by:

- A

  Abnormal left atrial contraction
- B

  Abnormal mitral valve closure
- C

  Abnormal aortic valve opening

:   **INCORRECT**

    Left atrial contraction occurs in diastole. Try again.
:   **CORRECT**

    Her mid-systolic click was caused by mitral valve prolapse without mitral regurgitation. Move on.
:   **INCORRECT**

    Please move on.
