# Supplementary material for: Professional Skill Builder: Mastering Cardiac Auscultation in Under 4 Hours
Source: MedEdPORTAL. 2017 May 8;13:10577. doi: 10.15766/mep_2374-8265.10577 (PMC6338255; doi:10.15766/mep_2374-8265.10577)
Supplement: Supplementary file 1 — A. PSB Mastering Cardiac Auscultation folder [file mep-13-10577-s001.zip › A. PSB Mastering Cardiac Auscultation folder/psb-mastering-cardiac-auscultation/assets/repo/cards_mini_case_06/c_06_4.html]

Professional Skill Builder | Cardiology Case 6


Cardiology Case 6

:   Next
:   Previous
:   (screen 4 of 4)

#### Summary:

This patient has a loud mid-sytolic click which is due to mitral valve prolapse. The click will be heard later in systole with squatting. Her mitral valve abnormality was confirmed by echocardiography**.**

*Click
to review auscultation.*

#### Lessons:

1. Her palpitation is suggestive of benign premature beats which may be totally unrelated to her MVP. You might suggest that she begin by limiting her coffee intake (decaf). If symptoms persist, consider getting a 24-hour ambulatory ECG.
2. Remember, mitral valve prolapse WILL worsen with time and she should be advised to have a heart examination about every three years. Sooner or later she will develop a late systolic murmur due to mitral regurgitation.

#### *Case 6 is now finished.*
