# Supplementary material for: Professional Skill Builder: Mastering Cardiac Auscultation in Under 4 Hours
Source: MedEdPORTAL. 2017 May 8;13:10577. doi: 10.15766/mep_2374-8265.10577 (PMC6338255; doi:10.15766/mep_2374-8265.10577)
Supplement: Supplementary file 1 — A. PSB Mastering Cardiac Auscultation folder [file mep-13-10577-s001.zip › A. PSB Mastering Cardiac Auscultation folder/psb-mastering-cardiac-auscultation/assets/repo/cards_mini_case_07/c_07_1.html]

Professional Skill Builder | Cardiology Case 7


Cardiology Case 7

:   Next
:   Previous
:   (screen 1 of 5)

**Instructions:**  After reading the patient summary, click
on the stethoscope head to listen to the heart sound. Then click on the
diagram that best matches what you hear.

A 17-year-old male high school student is seen for an athletic physical. No symptoms. **PMH**. negative (measles and chicken pox). **FH**. negative. **SH**. varsity soccer player. **Physical
Examination:** BP 140/60, P 60, R 12, Ht. 5'10", Wt. 175. General examination negative. Carotid pulses visible with brisk upstroke. No cardiac enlargement. Apex impulse quite forceful but not sustained. The following was heard on auscultation

AORTIC AREA, DIAPHRAGM (Sitting)

:   **INCORRECT**

    This patient does not have a mid-systolic click of late systolic murmur. The heart sound matching this diagram is playing now. Please try again.
:   **INCORRECT**

    The patient does not have a systolic murmur or S_3_. The heart sound matching this diagram is playing now. Please try again.
:   **CORRECT**

    Play the phono and then, if you wish, click on the incorrect diagrams
    to compare heart sounds.

    Move on.

- Play
  Phono
