# Supplementary material for: Professional Skill Builder: Mastering Cardiac Auscultation in Under 4 Hours
Source: MedEdPORTAL. 2017 May 8;13:10577. doi: 10.15766/mep_2374-8265.10577 (PMC6338255; doi:10.15766/mep_2374-8265.10577)
Supplement: Supplementary file 1 — A. PSB Mastering Cardiac Auscultation folder [file mep-13-10577-s001.zip › A. PSB Mastering Cardiac Auscultation folder/psb-mastering-cardiac-auscultation/assets/repo/cards_mini_case_07/c_07_2.html]

Professional Skill Builder | Cardiology Case 7


Cardiology Case 7

:   Next
:   Previous
:   (screen 2 of 5)

**Instructions:**  Review the patient's auscultation and
answer the question below.

*Click
to review auscultation.*

##### This patient has:

- A

  A click and a high-pitched systolic murmur
- B

  A click and a high-pitched diastolic murmur
- C

  An S_3_

:   **INCORRECT**

    Try again.
:   **CORRECT**

    Move on to the next screen.
:   **INCORRECT**

    Select again.
