# Supplementary material for: Professional Skill Builder: Mastering Cardiac Auscultation in Under 4 Hours
Source: MedEdPORTAL. 2017 May 8;13:10577. doi: 10.15766/mep_2374-8265.10577 (PMC6338255; doi:10.15766/mep_2374-8265.10577)
Supplement: Supplementary file 1 — A. PSB Mastering Cardiac Auscultation folder [file mep-13-10577-s001.zip › A. PSB Mastering Cardiac Auscultation folder/psb-mastering-cardiac-auscultation/assets/repo/cards_mini_case_07/c_07_3.html]

Professional Skill Builder | Cardiology Case 7


Cardiology Case 7

:   Next
:   Previous
:   (screen 3 of 5)

**Instructions:** The click here is subtle and is heard right after S_1_. It sounds like a split S_1_, but S_1_ splitting is never heard in this location (aortic area). It is called an ejection click (EC), and it originates from the opening of the aortic valve. Listen again and make sure you hear it.

Move on to the next screen when done.

Play Phono
