# Supplementary material for: Professional Skill Builder: Mastering Cardiac Auscultation in Under 4 Hours
Source: MedEdPORTAL. 2017 May 8;13:10577. doi: 10.15766/mep_2374-8265.10577 (PMC6338255; doi:10.15766/mep_2374-8265.10577)
Supplement: Supplementary file 1 — A. PSB Mastering Cardiac Auscultation folder [file mep-13-10577-s001.zip › A. PSB Mastering Cardiac Auscultation folder/psb-mastering-cardiac-auscultation/assets/repo/cards_mini_case_07/c_07_4.html]

Professional Skill Builder | Cardiology Case 7


Cardiology Case 7

:   Next
:   Previous
:   (screen 4 of 5)

**Instructions:**  Review the patient's auscultation and
answer the questions below.

*Click
to review auscultation.*

##### In this patient (17-year-old athlete) these findings would be considered:

- A

  Definitely abnormal
- B

  Probably abnormal
- C

  Probably normal

:   **CORRECT**

    A diastolic murmur is never "innocent" - it is always "guilty".

    Show Next Question
:   **INCORRECT**

    Try again.
:   **INCORRECT**

    Select again.

*Click
to review auscultation.*

##### It is probably caused by:

- A

  Aortic stenosis
- B

  Mitral valve prolapse
- C

  Aortic regurgitation

:   **INCORRECT**

    Try again.
:   **INCORRECT**

    Select again.
:   **CORRECT**

    Move on to the next screen.
