# Supplementary material for: Professional Skill Builder: Mastering Cardiac Auscultation in Under 4 Hours
Source: MedEdPORTAL. 2017 May 8;13:10577. doi: 10.15766/mep_2374-8265.10577 (PMC6338255; doi:10.15766/mep_2374-8265.10577)
Supplement: Supplementary file 1 — A. PSB Mastering Cardiac Auscultation folder [file mep-13-10577-s001.zip › A. PSB Mastering Cardiac Auscultation folder/psb-mastering-cardiac-auscultation/assets/repo/cards_mini_case_07/c_07_5.html]

Professional Skill Builder | Cardiology Case 7


Cardiology Case 7

:   Next
:   Previous
:   (screen 5 of 5)

#### Summary:

Every year, mild valvular heart disease is identified by vigilant physicians on routine physical exams. In this case, an echo was obtained which confirmed the presence of mild aortic regurgitation due to a congenitally bicuspid aortic valve.

*Click
to review auscultation.*

#### Lessons:

1. The ejection click here favors a congenital basis for this student's aortic regurgitation. It is produced by the opening of a deformed aortic valve (like the popping open of a parachute).
2. Follow up is crucial. Every two years is usually adequate at first.
3. If it is severe he/she should be referred to a cardiologist immediately and advised to hold off on competitive sports in the mean time.

#### *Case 7 is now finished.*
