# Supplementary material for: Professional Skill Builder: Mastering Cardiac Auscultation in Under 4 Hours
Source: MedEdPORTAL. 2017 May 8;13:10577. doi: 10.15766/mep_2374-8265.10577 (PMC6338255; doi:10.15766/mep_2374-8265.10577)
Supplement: Supplementary file 1 — A. PSB Mastering Cardiac Auscultation folder [file mep-13-10577-s001.zip › A. PSB Mastering Cardiac Auscultation folder/psb-mastering-cardiac-auscultation/assets/repo/cards_mini_case_08/c_08_1.html]

Professional Skill Builder | Cardiology Case 8


Cardiology Case 8

:   Next
:   Previous
:   (screen 1 of 5)

**Instructions:**  After reading the patient summary, click
on the stethoscope head to listen to the heart sound. Then click on the
diagram that best matches what you hear.

A 27-year-old male with unexplained fever, malaise and weakness of two weeks duration. **PMH**. Known systolic murmur; recent upper respiratory infection with severe bronchitis.**FH**. negative. **SH**. Non-smoker. **Physical
Examination:** BP 110/70, P 88, R 16, T 101° Carotid and jugular pulses normal. Lungs clear. No cardiac enlargement. The following was heard at the apex (see picture).

APEX, BELL (Left decubitus)

:   **CORRECT**

    Play the phono and then, if you wish, click on the incorrect diagrams
    to compare heart sounds.

    Move on.
:   **INCORRECT**

    There is a systolic murmur but there is no S_3_. The heart sound matching this diagram is playing now. Please try again.
:   **INCORRECT**

    There is a systolic murmur but there is no diastolic murmur. The heart sound matching this diagram is playing now. Please try again.

- Play
  Phono
