# Supplementary material for: Professional Skill Builder: Mastering Cardiac Auscultation in Under 4 Hours
Source: MedEdPORTAL. 2017 May 8;13:10577. doi: 10.15766/mep_2374-8265.10577 (PMC6338255; doi:10.15766/mep_2374-8265.10577)
Supplement: Supplementary file 1 — A. PSB Mastering Cardiac Auscultation folder [file mep-13-10577-s001.zip › A. PSB Mastering Cardiac Auscultation folder/psb-mastering-cardiac-auscultation/assets/repo/cards_mini_case_08/c_08_3.html]

Professional Skill Builder | Cardiology Case 8


Cardiology Case 8

:   Next
:   Previous
:   (screen 3 of 5)

#### Isometric Handgrip for Suspected Mitral Regurgitation

Isometric exercise is applied, in this case, by having the patient squeeze
and pull on a rolled towel. Usually it takes about 30 seconds for the
hemodynamic effect to manifest itself. Note that the heart rate increases.

**Click on the Play Phono buttons below to see and hear this phenomenon for
yourself. Move on when finished.**

Control
:   Play Phono


After 30 seconds of isometric handgrip
:   Play Phono
