# Supplementary material for: Professional Skill Builder: Mastering Cardiac Auscultation in Under 4 Hours
Source: MedEdPORTAL. 2017 May 8;13:10577. doi: 10.15766/mep_2374-8265.10577 (PMC6338255; doi:10.15766/mep_2374-8265.10577)
Supplement: Supplementary file 1 — A. PSB Mastering Cardiac Auscultation folder [file mep-13-10577-s001.zip › A. PSB Mastering Cardiac Auscultation folder/psb-mastering-cardiac-auscultation/assets/repo/cards_mini_case_08/c_08_4.html]

Professional Skill Builder | Cardiology Case 8


Cardiology Case 8

:   Next
:   Previous
:   (screen 4 of 5)

**Instructions:**  Answer the question below.

##### Isometric Handgrip for Suspected Mitral Regurgitation

##### The increase in the loudness of this patient's systolic murmur favors a diagnosis of:

- A

  Aortic stenosis
- B

  Tricuspid regurgitation
- C

  Mitral regurgitation

:   **INCORRECT**

    Try again.
:   **INCORRECT**

    Try another answer.
:   **CORRECT**

    With isometric exercise there is an increase in sympatheltic tone with elevation of mean blood pressure, as well as an increase in heart rate. The increase in afterload favors an increase in the mitral regurgitation that intensifies the murmur in about sixty-five percent of patients.

    **Click on the PHONOCARDIOGRAMS to compare the loudness of the heart murmurs again.**

    Note: The murmur of ventricular sepetal defect also intensifies with isometric exercise but is rarely apical.
