# Supplementary material for: Professional Skill Builder: Mastering Cardiac Auscultation in Under 4 Hours
Source: MedEdPORTAL. 2017 May 8;13:10577. doi: 10.15766/mep_2374-8265.10577 (PMC6338255; doi:10.15766/mep_2374-8265.10577)
Supplement: Supplementary file 1 — A. PSB Mastering Cardiac Auscultation folder [file mep-13-10577-s001.zip › A. PSB Mastering Cardiac Auscultation folder/psb-mastering-cardiac-auscultation/assets/repo/cards_mini_case_08/c_08_5.html]

Professional Skill Builder | Cardiology Case 8


Cardiology Case 8

:   Next
:   Previous
:   (screen 5 of 5)

#### Summary:

This patient was aware of a systolic murmur. Blood cultures were positive
for Strep viridans. An echocardiogram confirmed the presence of mitral regurgitation and showed vegetations on the mitral leaflets.

*Click
to review auscultation.*

#### Lessons:

1. The location, quality, and loudness of this murmur were important in
   considering mitral regurgitation as the cause.
2. Transesophageal echocardiography is often the best way to evaluate the possibility of valvular vegetations.In this case this was not necessary because the trans-thoracic echo had demonstrated vegetations. These abnormalities will disappear with antibiotic therapy.
3. Once the infection is cured, he should be able to resume full activity but he should be advised to have routine follow up evaluations every two years.

#### *Case 8 is now finished.*
