# Supplementary material for: Professional Skill Builder: Mastering Cardiac Auscultation in Under 4 Hours
Source: MedEdPORTAL. 2017 May 8;13:10577. doi: 10.15766/mep_2374-8265.10577 (PMC6338255; doi:10.15766/mep_2374-8265.10577)
Supplement: Supplementary file 1 — A. PSB Mastering Cardiac Auscultation folder [file mep-13-10577-s001.zip › A. PSB Mastering Cardiac Auscultation folder/psb-mastering-cardiac-auscultation/assets/repo/cards_mini_case_09/c_09_1.html]

Professional Skill Builder | Cardiology Case 9


Cardiology Case 9

:   Next
:   Previous
:   (screen 1 of 4)

**Instructions:**  After reading the patient summary, click
on the stethoscope head to listen to the heart sound. Then click on the
diagram that best matches what you hear.

A 60-year-old police officer with recent onset of mild exertional dyspnea associated with moderate substernal discomfort. Quickly relieved by rest. **Vitals: BP** 116/80, **P** 76**, R** 12, **Wt.** 192, **Ht.** 72 inches **PMH**. Questionable rheumatic fever as a child. Known heart murmur for 20 years. **FH**. Negative for CV disease. **SH**. Smokes occasional cigar. **Physical
Examination:** Slow-rising carotid upstrokes. Bilateral carotid bruits. No venous distension. Lungs clear. No cardiac enlargement. The apex impulse is sustained consistent with left ventricular hypertrophy. The following was present on auscultation.

AORTIC AREA, DIAPHRAGM (Supine)

:   **INCORRECT**

    This patient does not have a diastolic murmur. The heart sound matching this diagram is playing now. Please try again.
:   **INCORRECT**

    This patient does not have an audible S_2_. The heart sound matching this diagram is playing now. Try again.
:   **CORRECT**

    Play the phono and then, if you wish, click on the incorrect diagrams
    to compare heart sounds.

    Move on to the next screen.

- Play
  Phono
