# Supplementary material for: Professional Skill Builder: Mastering Cardiac Auscultation in Under 4 Hours
Source: MedEdPORTAL. 2017 May 8;13:10577. doi: 10.15766/mep_2374-8265.10577 (PMC6338255; doi:10.15766/mep_2374-8265.10577)
Supplement: Supplementary file 1 — A. PSB Mastering Cardiac Auscultation folder [file mep-13-10577-s001.zip › A. PSB Mastering Cardiac Auscultation folder/psb-mastering-cardiac-auscultation/assets/repo/cards_mini_case_09/c_09_2.html]

Professional Skill Builder | Cardiology Case 9


Cardiology Case 9

:   Next
:   Previous
:   (screen 2 of 4)

**Instructions:**  Review the patient's auscultation and
answer the questions below.

*Click
to review auscultation.*

##### This patient's murmur is best described as:

- A

  Systolic ejection
- B

  Holosystolic regurgitant
- C

  Late systolic

:   **CORRECT**

    Show Next Question
:   **INCORRECT**

    Select again.
:   **INCORRECT**

    Try again.

*Click
to review auscultation.*

##### Normally S_2_ in this location (the aortic area) is caused by:

- A

  Pulmonary valve closure
- B

  Aortic valve closure
- C

  Both aortic and pulmonary closure

:   **INCORRECT**

    Try again.
:   **CORRECT**

    Move on to the next screen.
:   **INCORRECT**

    Although rarely, pulmonary valve closure is audible in the aortic area.

    Select again.
