# Supplementary material for: Professional Skill Builder: Mastering Cardiac Auscultation in Under 4 Hours
Source: MedEdPORTAL. 2017 May 8;13:10577. doi: 10.15766/mep_2374-8265.10577 (PMC6338255; doi:10.15766/mep_2374-8265.10577)
Supplement: Supplementary file 1 — A. PSB Mastering Cardiac Auscultation folder [file mep-13-10577-s001.zip › A. PSB Mastering Cardiac Auscultation folder/psb-mastering-cardiac-auscultation/assets/repo/cards_mini_case_09/c_09_3.html]

Professional Skill Builder | Cardiology Case 9


Cardiology Case 9

:   Next
:   Previous
:   (screen 3 of 4)

**Instructions:**  Review the patient's auscultation and
answer the question below.

*Click
to review auscultation.*

##### The absence of S_2_ would favor:

- A

  Calcific aortic stenosis with impaired cusp mobility
- B

  Severe aortic regurgitation
- C

  Mitral stenosis

:   **CORRECT**

    With a heavily calcified aortic valve, S_2_ is often very soft or absent.

    Move on to the next screen.
:   **INCORRECT**

    Try again.
:   **INCORRECT**

    Select again.
