# Supplementary material for: Professional Skill Builder: Mastering Cardiac Auscultation in Under 4 Hours
Source: MedEdPORTAL. 2017 May 8;13:10577. doi: 10.15766/mep_2374-8265.10577 (PMC6338255; doi:10.15766/mep_2374-8265.10577)
Supplement: Supplementary file 1 — A. PSB Mastering Cardiac Auscultation folder [file mep-13-10577-s001.zip › A. PSB Mastering Cardiac Auscultation folder/psb-mastering-cardiac-auscultation/assets/repo/cards_mini_case_09/c_09_4.html]

Professional Skill Builder | Cardiology Case 9


Cardiology Case 9

:   Next
:   Previous
:   (screen 4 of 4)

#### Summary:

This patient's echocardiogram demonstrated moderate LVH and calcific aortic stenosis with a mean gradient of 40 mm Hg (moderately severe).

*Click
to review auscultation.*

#### Lessons:

1. This patient's coronary arteries were **normal** on cardiac catheterization. His angina was due **entirely** to his aortic stenosis.
2. The carotid bruits, the slow carotid upstrokes, and the abnormal apex impulse, all pointed to significant aortic valve stenosis even before auscultation of the heart and echocardiography.
3. Because he now has a bioprosthesis in the aortic position, he requires antibiotic prophylaxis against infective endocarditis, but he does not require anticoagulant therapy. He has also been transferred to a desk job in the police department.

#### *Case 9 is now finished.*
