# Supplementary material for: Professional Skill Builder: Mastering Cardiac Auscultation in Under 4 Hours
Source: MedEdPORTAL. 2017 May 8;13:10577. doi: 10.15766/mep_2374-8265.10577 (PMC6338255; doi:10.15766/mep_2374-8265.10577)
Supplement: Supplementary file 1 — A. PSB Mastering Cardiac Auscultation folder [file mep-13-10577-s001.zip › A. PSB Mastering Cardiac Auscultation folder/psb-mastering-cardiac-auscultation/assets/repo/cards_mini_case_11/c_11_1.html]

Professional Skill Builder | Case 11


Cardiology Case 11

:   Next
:   Previous
:   (screen 1 of 4)

**Instructions:**  After reading the patient summary, click
on the stethoscope head to listen to the heart sound. Then click on the
diagram that best matches what you hear.

A 52-year-old auto worker is admitted to CCU with a large, anterolateral
myocardial infarction. He is moderately dyspneic. **FH**. Positive for CAD
and diabetes. **SH**. 1 1/2 pack per day smoker. **PMH**. Negative. **Physical Examination:** BP 100/60, P 108+reg., R 20. Normal carotid and jugular pulses. Bilateral
medium crackles posteriorly. No cardiac enlargement. The following was
present on auscultation.

APEX, BELL, (Left Decubitus)

:   **INCORRECT**

    Splitting of S_2_ is rarely audible at the apex. The heart sound matching
    this diagram is playing now. Please try again.
:   **CORRECT**

    Play the phono and then, if you wish, click on the incorrect diagrams
    to compare heart sounds.

    Move on to the next screen.
:   **INCORRECT**

    The patient does not have an S_4_. The heart sound matching this diagram is playing now. Please try again.

- Play
  Phono
