# Supplementary material for: Professional Skill Builder: Mastering Cardiac Auscultation in Under 4 Hours
Source: MedEdPORTAL. 2017 May 8;13:10577. doi: 10.15766/mep_2374-8265.10577 (PMC6338255; doi:10.15766/mep_2374-8265.10577)
Supplement: Supplementary file 1 — A. PSB Mastering Cardiac Auscultation folder [file mep-13-10577-s001.zip › A. PSB Mastering Cardiac Auscultation folder/psb-mastering-cardiac-auscultation/assets/repo/cards_mini_case_11/c_11_4.html]

Professional Skill Builder | Case 11


Cardiology Case 11

:   Next
:   Previous
:   (screen 4 of 4)

#### Summary:

On the basis of her clinical examination, the house officer placed a Swan-Ganz
catheter which showed acute left ventricular failure. PAW 28 mm Hg. C.O.
2.85 L/min. The patient proceeded to develop cardiogenic shock and ended
up having urgent cardiac catheterization and angioplasty of his infarct
vessel. He survived.

*Click
to review auscultation.*

#### Lessons:

1. An S_3_ gallop in the setting of an acute MI is important and has prognostic
   and therapeutic significance.
2. It usually indicates at least moderate left ventricular dysfunction.
3. It will often be heard only if properly sought after. Remember the
   word BALLS. (Use your Bell,
   at the Apex, with very Light
   pressure, patient on their Left Side).
4. Remember the cadence which sounds like: "I'm dying. I'm dying. I'm
   dying." " Please help me! Please help me! Please help me!"

#### *Case 11 is now finished.*
