# Supplementary material for: Professional Skill Builder: Mastering Cardiac Auscultation in Under 4 Hours
Source: MedEdPORTAL. 2017 May 8;13:10577. doi: 10.15766/mep_2374-8265.10577 (PMC6338255; doi:10.15766/mep_2374-8265.10577)
Supplement: Supplementary file 1 — A. PSB Mastering Cardiac Auscultation folder [file mep-13-10577-s001.zip › A. PSB Mastering Cardiac Auscultation folder/psb-mastering-cardiac-auscultation/assets/repo/cards_mini_case_13/c_13_1.html]

Professional Skill Builder | Cardiology Case 13


Cardiology Case 13

:   Next
:   Previous
:   (screen 1 of 10)

**Instructions:**  After reading the patient summary, click
on the stethoscope head to listen to the heart sound. Then click on the
diagram that best matches what you hear.

A 22-year-old female law student with chest pain. Pain usually comes on while studying. It is sharp, aching, recurrent and localized around the left scapula. Started 3 months ago. Lasts seconds at a time.
**Physical Examination:** BP108/72, P 76, R 12. Her exam is entirely normal except for this finding.

AORTCI AREA, DIAPHRAGM (Sitting)

:   **Yes,**

    but there is more. The heart sound matching this diagram is playing now. Please try again.
:   **INCORRECT**

    The patient does not have a diastolic murmur. The heart sound matching this diagram is playing now. Please try again.
:   **CORRECT**

    Play the phono and then, if you wish, click on the incorrect diagrams
    to compare heart sounds.

    Move on to the next screen.

- Play
  Phono
