# Supplementary material for: Professional Skill Builder: Mastering Cardiac Auscultation in Under 4 Hours
Source: MedEdPORTAL. 2017 May 8;13:10577. doi: 10.15766/mep_2374-8265.10577 (PMC6338255; doi:10.15766/mep_2374-8265.10577)
Supplement: Supplementary file 1 — A. PSB Mastering Cardiac Auscultation folder [file mep-13-10577-s001.zip › A. PSB Mastering Cardiac Auscultation folder/psb-mastering-cardiac-auscultation/assets/repo/cards_mini_case_13/c_13_10.html]

Professional Skill Builder | Cardiology Case 13


Cardiology Case 13

:   Next
:   Previous
:   (screen 10 of 10)

#### Summary:

This patient's mitral valve prolapse was confirmed by echocardiography.

*Click
to review auscultation.*

#### Lessons:

1. Although her chest pain was probably unrelated to her MVP, she was fortunate that it brought her to a physician and that her mitral valve abnormality was discovered (the pain responded to acetaminophen).
2. She was advised to return for a check-up every 2-3 years.
3. No restriction whatsoever on physical activity.
4. Since the deformed mitral valve which causes the prolapse is hereditary, it was recommended that her parents and siblings be checked for possible MVP.

**Note:** Repeat echocardiogram will only be necessary if patient becomes symptomatic or possibly in about 5 years.

#### *Case 13 is now finished.*
