# Supplementary material for: Professional Skill Builder: Mastering Cardiac Auscultation in Under 4 Hours
Source: MedEdPORTAL. 2017 May 8;13:10577. doi: 10.15766/mep_2374-8265.10577 (PMC6338255; doi:10.15766/mep_2374-8265.10577)
Supplement: Supplementary file 1 — A. PSB Mastering Cardiac Auscultation folder [file mep-13-10577-s001.zip › A. PSB Mastering Cardiac Auscultation folder/psb-mastering-cardiac-auscultation/assets/repo/cards_mini_case_13/c_13_2.html]

Professional Skill Builder | Cardiology Case 13


Cardiology Case 13

:   Next
:   Previous
:   (screen 2 of 10)

**Instructions:**  Review the patient's auscultation and
answer the questions below.

*Click
to review auscultation.*

##### This patient has:

- A

  A systolic murmur
- B

  A systolic click and a systolic murmur
- C

  A diastolic murmur

:   **INCORRECT**

    Yes, but there is more than a murmur.

    Try again.
:   **CORRECT**

    She has both a systolic click and a systolic murmur.

    Show Next Question
:   **INCORRECT**

    She has no diastolic murmur.

    Select again.

*Click
to review auscultation.*

##### In this location (apex), it is probably due to:

- A

  Aortic stenosis
- B

  Mitral valve prolapse with mitral regurgitation
- C

  Innocent murmur

:   **INCORRECT**

    Try again.
:   **CORRECT**

    This is due to mitral valve prolapse.

    Move on to the next screen.
:   **INCORRECT**

    It is unlikely that this is an innocent murmur.

    Select again.
