# Supplementary material for: Professional Skill Builder: Mastering Cardiac Auscultation in Under 4 Hours
Source: MedEdPORTAL. 2017 May 8;13:10577. doi: 10.15766/mep_2374-8265.10577 (PMC6338255; doi:10.15766/mep_2374-8265.10577)
Supplement: Supplementary file 1 — A. PSB Mastering Cardiac Auscultation folder [file mep-13-10577-s001.zip › A. PSB Mastering Cardiac Auscultation folder/psb-mastering-cardiac-auscultation/assets/repo/cards_mini_case_13/c_13_4.html]

Professional Skill Builder | Cardiology Case 13


Cardiology Case 13

:   Next
:   Previous
:   (screen 4 of 10)

#### Prompt Squatting Maneuver 2

Play instructions

Move on to the next screen when done.
