# Supplementary material for: Professional Skill Builder: Mastering Cardiac Auscultation in Under 4 Hours
Source: MedEdPORTAL. 2017 May 8;13:10577. doi: 10.15766/mep_2374-8265.10577 (PMC6338255; doi:10.15766/mep_2374-8265.10577)
Supplement: Supplementary file 1 — A. PSB Mastering Cardiac Auscultation folder [file mep-13-10577-s001.zip › A. PSB Mastering Cardiac Auscultation folder/psb-mastering-cardiac-auscultation/assets/repo/cards_mini_case_17/c_17_1.html]

Professional Skill Builder | Cardiology Case 17


Cardiology Case 17

:   Next
:   Previous
:   (screen 1 of 4)

**Instructions:**  After reading the patient summary, click
on the stethoscope head to listen to the heart sound. Then click on the
diagram that best matches what you hear.

A 30-year-old teacher is seen because of a family history of mitral valve prolapse (mother). She has no symptoms but is concerned because she has been told it is hereditary. **Physical Examination:** BP 114/70, P 66+reg, R 12. General examination entirely negative. The following was heard on auscultation.

TRICUSPID AREA, BELL (Supine)

:   **INCORRECT**

    This patient does not have a mid-systolic click. The heart sound matching this diagram is playing now. Please try again.
:   **INCORRECT**

    This patient does not have an S_4_. The heart sound matching this diagram is playing now. Please try again.
:   **CORRECT**

    Play the phono and then, if you wish, click on the incorrect diagrams
    to compare heart sounds.

    Move on.

- Play
  Phono
