# Supplementary material for: Professional Skill Builder: Mastering Cardiac Auscultation in Under 4 Hours
Source: MedEdPORTAL. 2017 May 8;13:10577. doi: 10.15766/mep_2374-8265.10577 (PMC6338255; doi:10.15766/mep_2374-8265.10577)
Supplement: Supplementary file 1 — A. PSB Mastering Cardiac Auscultation folder [file mep-13-10577-s001.zip › A. PSB Mastering Cardiac Auscultation folder/psb-mastering-cardiac-auscultation/assets/repo/cards_mini_case_17/c_17_2.html]

Professional Skill Builder | Cardiology Case 17


Cardiology Case 17

:   Next
:   Previous
:   (screen 2 of 4)

**Instructions:**  Review the patient's auscultation and
answer the questions below.

*Click
to review heart sound.*

##### This patient has:

- A

  A systolic click
- B

  A split S_1_
- C

  An S_4_

:   **INCORRECT**

    Try again.
:   **CORRECT**

    Be sure to note the location of the stethoscope on this patient. It is in the tricuspid area.

    Show Next Question
:   **INCORRECT**

    Select again.

*Click
to review heart sound.*

##### In this location it is:

- A

  Normal
- B

  Probably due to to mitral valve prolapse
- C

  Probably due to left bundle branch block

:   **CORRECT**

    In this location 85% of normal people have a split S_1_.  
    Move on to the next screen.
:   **INCORRECT**

    Try again.
:   **INCORRECT**

    A split S_1_ is normal although it can occur with bundle branch block.

    Select again.
