# Supplementary material for: Professional Skill Builder: Mastering Cardiac Auscultation in Under 4 Hours
Source: MedEdPORTAL. 2017 May 8;13:10577. doi: 10.15766/mep_2374-8265.10577 (PMC6338255; doi:10.15766/mep_2374-8265.10577)
Supplement: Supplementary file 1 — A. PSB Mastering Cardiac Auscultation folder [file mep-13-10577-s001.zip › A. PSB Mastering Cardiac Auscultation folder/psb-mastering-cardiac-auscultation/assets/repo/cards_mini_case_17/c_17_3.html]

Professional Skill Builder | Cardiology Case 17


Cardiology Case 17

:   Next
:   Previous
:   (screen 3 of 4)

**Instructions:**  Review the patient's auscultation and
answer the questions below.

*Click
to review heart sound.*

##### This split S_1_ is due to:

- A

  Asynchronous tricuspid valve closure and pulmonic
  valve opening.
- B

  Asynchronous tricuspid valve closure and right
  atrial contraction.
- C

  Asynchronous tricuspid valve closure and mitral
  valve closure.

:   **INCORRECT**

    Pulmonic valve opening is normally inaudible.

    Try again.
:   **INCORRECT**

    Right atrial contraction is normally inaudible.

    Select again.

    :   **CORRECT**

        Show Next Question

*Click
to review heart sound.*

##### Which closes first?

- A

  Mitral valve
- B

  Tricuspid valve

:   **CORRECT**

    Move on to the next screen.
:   **INCORRECT**

    Select again.
