# Supplementary material for: Professional Skill Builder: Mastering Cardiac Auscultation in Under 4 Hours
Source: MedEdPORTAL. 2017 May 8;13:10577. doi: 10.15766/mep_2374-8265.10577 (PMC6338255; doi:10.15766/mep_2374-8265.10577)
Supplement: Supplementary file 1 — A. PSB Mastering Cardiac Auscultation folder [file mep-13-10577-s001.zip › A. PSB Mastering Cardiac Auscultation folder/psb-mastering-cardiac-auscultation/assets/repo/cards_mini_case_17/c_17_4.html]

Professional Skill Builder | Cardiology Case 17


Cardiology Case 17

:   Next
:   Previous
:   (screen 4 of 4)

//

#### Summary:

Because of the patient's concern, an echo study was performed that showed no evidence of mitral valve abnormality.

*Click
to review auscultation.*

#### Lessons:

1. Splitting of S_1_ is normal and must not be confused with the systolic click of mitral valve prolapse.
2. Splitting of S_1_ is almost universal in the tricuspid area but is also **often present at the apex** in perfectly normal people.
3. When in doubt have the patient squat.  
   a. A split S1 does not change.  
   b. A systolic click due to MVP moves later in systole and becomes more mid-systolic in cadence.
4. Review Case 4 on how to differentiate a split S_1_ from an S_4_.

#### *Case 17 is now finished.*
