# Supplementary material for: Professional Skill Builder: Mastering Cardiac Auscultation in Under 4 Hours
Source: MedEdPORTAL. 2017 May 8;13:10577. doi: 10.15766/mep_2374-8265.10577 (PMC6338255; doi:10.15766/mep_2374-8265.10577)
Supplement: Supplementary file 1 — A. PSB Mastering Cardiac Auscultation folder [file mep-13-10577-s001.zip › A. PSB Mastering Cardiac Auscultation folder/psb-mastering-cardiac-auscultation/assets/repo/cards_mini_case_20/c_20_1.html]

Professional Skill Builder | Cardiology Case 20


Cardiology Case 20

:   Next
:   Previous
:   (screen 1 of 4)

**Instructions:**  After reading the patient summary, click
on the stethoscope head to listen to the heart sound. Then click on the
diagram that best matches what you hear.

A 43-year-old house painter is admitted because of progressive dyspnea, orthopnea and edema. He is alcoholic and has had one previous hospitalization for acute alcoholism. **Physical
Examination:** BP 112/80, P 94+reg., R 24. Moderate signs of congestive failure with jugular venous distension, hepatomegaly, 2+ edema, and bilateral crackles. The heart border is 12 cm from the midsternal line with a diffuse sustained apical impulse. The following was heard on auscultation.

APEX, BELL (Left Decubitus)

:   **INCORRECT**

    Correct but there is more.The heart sound matching this diagram is playing now. Please try again.
:   **CORRECT**

    Play the phono and then, if you wish, click on the incorrect diagrams
    to compare heart sounds.

    Move on.
:   **INCORRECT**

    This patient does not have a diastolic murmur. The heart sound matching this diagram is playing now. Please try again.

- Play
  Phono
