# Supplementary material for: Professional Skill Builder: Mastering Cardiac Auscultation in Under 4 Hours
Source: MedEdPORTAL. 2017 May 8;13:10577. doi: 10.15766/mep_2374-8265.10577 (PMC6338255; doi:10.15766/mep_2374-8265.10577)
Supplement: Supplementary file 1 — A. PSB Mastering Cardiac Auscultation folder [file mep-13-10577-s001.zip › A. PSB Mastering Cardiac Auscultation folder/psb-mastering-cardiac-auscultation/assets/repo/cards_mini_case_20/c_20_2.html]

Professional Skill Builder | Cardiology Case 20


Cardiology Case 20

:   Next
:   Previous
:   (screen 2 of 4)

**Instructions:**  Review the patient's auscultation and
answer the questions below.

*Click
to review auscultation.*

##### This patient has:

- A

  A systolic murmur and a split S_2_
- B

  A systolic murmur and a diastolic murmur
- C

  A systolic murmur and an S_3_

:   **INCORRECT**

    A split S_2_ is almost never audible in this location (apex).

    Try again.
:   **INCORRECT**

    There is no diastolic murmur.

    Select again.
:   **CORRECT**

    Show Next Question

*Click
to review auscultation.*

##### The S_3_ is probably:

- A

  Physiologic and expected
- B

  Due to increased cardiac output
- C

  Due to poor LV function with a high diastolic filling pressure

:   **INCORRECT**

    Certainly not.

    Select again.
:   **INCORRECT**

    This patient's cardiac output is *decreased*.

    Try again.
:   **CORRECT**

    This is a pathologic gallop.

    Move on to the next screen.
