# Supplementary material for: Professional Skill Builder: Mastering Cardiac Auscultation in Under 4 Hours
Source: MedEdPORTAL. 2017 May 8;13:10577. doi: 10.15766/mep_2374-8265.10577 (PMC6338255; doi:10.15766/mep_2374-8265.10577)
Supplement: Supplementary file 1 — A. PSB Mastering Cardiac Auscultation folder [file mep-13-10577-s001.zip › A. PSB Mastering Cardiac Auscultation folder/psb-mastering-cardiac-auscultation/assets/repo/cards_mini_case_20/c_20_4.html]

Professional Skill Builder | Cardiology Case 20


Cardiology Case 20

:   Next
:   Previous
:   (screen 4 of 4)

#### Summary:

On echocardiography this patient had a moderately dilated left ventricle
with severe systolic dysfunction (EF 25%). His mitral valve was structurally
normal but there was 2+ (moderate) mitral regurgitation.

*Click
to review auscultation.*

#### Lessons:

1. The patient probably has alcoholic cardiomyopathy with secondary mitral regurgitation due to his dilated left ventricle.
2. His extra sound was actually shown to be a "summation gallop" by Doppler. That is, as the heart rate speeded up and diastole became shorter, left atrial contraction occurred earlier and earlier until it actually occurred during the period of passive filling, e.g., atrial contraction fell right on the S_3_, producing an augmented ("summation") sound.
3. With treatment for heart failure and abstinence from alcohol there was significant improvement in LV function and both the murmur and the gallop sound became almost inaudible.

#### *Case 20 is now finished.*
