# Supplementary material for: Professional Skill Builder: Mastering Cardiac Auscultation in Under 4 Hours
Source: MedEdPORTAL. 2017 May 8;13:10577. doi: 10.15766/mep_2374-8265.10577 (PMC6338255; doi:10.15766/mep_2374-8265.10577)
Supplement: Supplementary file 1 — A. PSB Mastering Cardiac Auscultation folder [file mep-13-10577-s001.zip › A. PSB Mastering Cardiac Auscultation folder/psb-mastering-cardiac-auscultation/assets/repo/cards_review_the_basics/car_basic01_03.html]

Professional Skill Builder | Review The Basics


**Instructions:**

- play
- pause
- stop

- full
  screen
- restore
  screen

Update Required To play the
media you will need to either update your browser to a recent version
or update your Flash
plugin.
