# Supplementary material for: Professional Skill Builder: Mastering Cardiac Auscultation in Under 4 Hours
Source: MedEdPORTAL. 2017 May 8;13:10577. doi: 10.15766/mep_2374-8265.10577 (PMC6338255; doi:10.15766/mep_2374-8265.10577)
Supplement: Supplementary file 1 — A. PSB Mastering Cardiac Auscultation folder [file mep-13-10577-s001.zip › A. PSB Mastering Cardiac Auscultation folder/psb-mastering-cardiac-auscultation/assets/repo/cards_review_the_basics/car_basic01_14.html]

Professional Skill Builder | Module Title Here


**Instructions:**

**Very Important!**

- The S_3_ or S_4_ are usually soft, low-pitched, and
  hard to hear.

- When listening for an S_3_ or S_4_ gallop you should:
  - Use the bell of your stethoscope
  - at the apex
  - with very little pressure
  - with the patient rolled on their left side

- So - when listening for an S_3_ or S_4_ gallop remember
  the word "BALLS"
  > **B** - Bell   
  > **A** - Apex   
  > **L** - Light pressure   
  > **LS** - Left Side

**BALLS** for gallops

**Rare exception:** The occasional gallop arising from the right ventricle
may be heard along the left sternal border.

>
