# Supplementary material for: Professional Skill Builder: Mastering Cardiac Auscultation in Under 4 Hours
Source: MedEdPORTAL. 2017 May 8;13:10577. doi: 10.15766/mep_2374-8265.10577 (PMC6338255; doi:10.15766/mep_2374-8265.10577)
Supplement: Supplementary file 1 — A. PSB Mastering Cardiac Auscultation folder [file mep-13-10577-s001.zip › A. PSB Mastering Cardiac Auscultation folder/psb-mastering-cardiac-auscultation/assets/repo/primer_challenge/primer_challenge_1.html]

Professional Skill Builder | The Challenge


Heart Sound Primer: The Challenge

:   Next
:   Previous
:   (screen 1 of 2)

**Instructions:** Read the following introduction and then
move on to the next screen to take the Heart Sound Challenge.

##### Mastering Heart Sounds: The Challenge

The major objective of this section of the Primer is to help you become confident about your ability to correctly identify each of these basic heart sound cadences.

It will also allow you to identify your deficiencies and remediate them yourself.

You will be presented with a random series of heart sounds and be asked to identify the cadence. You will receive immediate feedback and will be able to start over as often as you wish. Your goal will be to correctly identify twenty four heart sound cadences in a row (sink 24 baskets so to speak). Once you have achieved this or at least your best possible score, move on to the Cardiac Cases.

In the PSB Dashboard you will have the opportunity to review authentic clinical cases, taken from the practices of the cardiology faculty, where correct identification of these heart sound cadences was particularly helpful in managing the patient's problem.

Note: you can return to The Challenge anytime to practice developing your listening skills and/or get your 24 in a row.

Caveat: the ability to listen and identify heart sounds will provide a strong foundation for your clinical practice for years to come. Much like riding a bicycle, these are skills that will remain with you for the rest of your life.

Move on to the next screen.
