# Supplementary material for: Professional Skill Builder: Mastering Cardiac Auscultation in Under 4 Hours
Source: MedEdPORTAL. 2017 May 8;13:10577. doi: 10.15766/mep_2374-8265.10577 (PMC6338255; doi:10.15766/mep_2374-8265.10577)
Supplement: Supplementary file 1 — A. PSB Mastering Cardiac Auscultation folder [file mep-13-10577-s001.zip › A. PSB Mastering Cardiac Auscultation folder/psb-mastering-cardiac-auscultation/assets/repo/primer_challenge/primer_challenge_2.html]

Professional Skill Builder | The Challenge


Heart Sound Primer: The Challenge

:   Next
:   Previous
:   (screen 2 of 2)

**Instructions:** Click on the 'Click to Play' to start and correctly identify twenty four heart sound cadences in a row.
