# Supplementary material for: Professional Skill Builder: Mastering Cardiac Auscultation in Under 4 Hours
Source: MedEdPORTAL. 2017 May 8;13:10577. doi: 10.15766/mep_2374-8265.10577 (PMC6338255; doi:10.15766/mep_2374-8265.10577)
Supplement: Supplementary file 1 — A. PSB Mastering Cardiac Auscultation folder [file mep-13-10577-s001.zip › A. PSB Mastering Cardiac Auscultation folder/psb-mastering-cardiac-auscultation/assets/repo/primer_heartsound/primer_heartsound.html]

Professional Skill Builder | Heart Sound & Murmur Library


Heart Sound & Murmur Library

:   (screen 1 of 1)

**Instructions:** Click on the diagram of the heartsound to listen to it or stop it from playing.

Download all sounds as mp3s

#### Apex Area - Supine, Listening with the bell of stethoscope

|  |  |  |  |
| --- | --- | --- | --- |
|  | **Single S1 S2**   Normal | **Split S1**    Normal | **Mid-Systolic Click**   Mitral Valve Prolapse |
|  |  |  |  |
| **Early Systolic Murmur**   Acute Mitral Regurgitation | **Mid-Systolic Murmur**   Mitral Regurgitation due to CAD (Coronary Artery Disease) | **Late Systolic Murmur**   Mitral Regurgitation due to MVP (Mitral Valve Prolapse) | **Holosystolic Murmur**   Classic Mitral Regurgitation -or- Ventricular Septal Defect when heard along the left sternal border |

#### Apex Area - Left Ducubitus, Listening with the bell of stethoscope

|  |  |  |  |
| --- | --- | --- | --- |
|  | **S4 Gallop**   Left Ventricular Hypertrophy | **S3 Gallop**    Both Normal and Cardiomyopathy | **Systolic Click with Late Systolic Murmur**   Mitral Valve Prolapse with Mitral Regurgitation |
|  |  |  |  |
| **S4 and Mid-Systolic Murmur**   Ischemic Cardiomyopathy with Mitral Regurgitation | **S3 and Holosystolic Murmur**   Dilated Cardiomyopathy with Mitral Regurgitation | **Mitral Opening Snap and Diastolic Murmur**   Mitral Stenosis |  |

#### Aortic Area - Sitting, Listening with the bell of stethoscope

|  |  |  |  |
| --- | --- | --- | --- |
|  | **Normal S1 S2**   Supine Normal | **Systolic Murmur with Absent S2**   Severe Aortic Stenosis | **Early Diastolic Murmur**   Aortic Regurgitation |
|  |  |  |  |
| **Systolic and Diastolic Murmurs**   Combined Aortic Stenosis and Regurgitation |  |  |  |

#### Pulmonic Area - Supine, Listening with the diaphragm of stethoscope

|  |  |  |  |
| --- | --- | --- | --- |
|  | **Single S2**   Normal in Elderly | **Split S2 Persistent**   Complete Right Bundle Branch Block (On ECG) | **Split S2 Transient**   Normal |
|  |  |  |  |
| **Ejection Systolic Murmur with  Transient Splitting S2**   Innocent Murmur | **Ejection Systolic Murmur with Persistent Split S2 and Ejection, Systolic Murmur**   Arterial Septal Defect | **Ejection Systolic Murmur with Single S2 and Ejection Click**   Pulmonary Valve Stenosis |  |
