# Supplementary material for: Professional Skill Builder: Mastering Cardiac Auscultation in Under 4 Hours
Source: MedEdPORTAL. 2017 May 8;13:10577. doi: 10.15766/mep_2374-8265.10577 (PMC6338255; doi:10.15766/mep_2374-8265.10577)
Supplement: Supplementary file 1 — A. PSB Mastering Cardiac Auscultation folder [file mep-13-10577-s001.zip › A. PSB Mastering Cardiac Auscultation folder/psb-mastering-cardiac-auscultation/assets/repo/primer_tutorial_v2/cards_primer_09.html]

Professional Skill Builder | Heart Sound Primer


:   Nav

**Instructions:**

:   **Listen Below:**

    Apex,
    Normal S1 S2
    Tricuspid,
    Split S1

:   **Listen Below:**

    Pulmonic,
    Split S2

:   **Listen Below:**

    Aortic, Normal S1 S2
    Aortic, Systolic Murmur
    Aortic, Diastolic Murmur

:   **Listen Below:**

    Left Decubitus, Apex, S3 Gallop
    Left Decubitus, Apex, S4 Gallop
