# Supplementary material for: Professional Skill Builder: Mastering Cardiac Auscultation in Under 4 Hours
Source: MedEdPORTAL. 2017 May 8;13:10577. doi: 10.15766/mep_2374-8265.10577 (PMC6338255; doi:10.15766/mep_2374-8265.10577)
Supplement: Supplementary file 1 — A. PSB Mastering Cardiac Auscultation folder [file mep-13-10577-s001.zip › A. PSB Mastering Cardiac Auscultation folder/psb-mastering-cardiac-auscultation/start-here.html]

Professional Skill Builder: Mastering Cardiac Auscultation in Under Four Hours


# Professional Skill Builder: Mastering Cardiac Auscultation in Under Four Hours


## About This Course

This course provides health science students a guided approach to develop their cardiac ausculation skills. Students who master the content in this module will have the ability and confidence to perform basic cardiac ausculation in the clinical setting.

The instructional focus is on teaching the eight basic heart cadences and their clinical significance through the use of tutorials, a practice challenge, and interactive cases. All of the materials in the program are authentic and were developed by academic cardiologists and internists.

## Audience

- Medical, nursing, dental, and physician assistant students.
- Practicing health care professionals.

#### Audio Requirements

- Use head phones or ear buds (sounds are quiet and difficult to hear over computer speakers).
- Try to match the loudness to that of your own heart. Over amplification will cause sound distortion.

## Course Content:

**1. Basics of Cardiac Auscultation** (10 min)

- Start here. Get oriented to your stethoscope and cardiac auscultation.

**2. Heart Sound Primer** (12 min)

- Now learn the eight basic cadences.

**3. Heart Sound Challenge** (45-90 min)

- Master the cadences. Play this "game" to make recognition of the sound cadences automatic by imprinting them in your "fast thinking".

**4. Clinical Cases** (11 cases, 10 min each)  
Show Cases 

- Apply your knowledge and skills. Practice on authentic patient cases.

  1. Normal cardiac exam in a patient with CAD
  2. CAD with MR and S4
  3. SBE in a patient with chronic MR
  4. Bicuspid aortic valve with AR in a 17 year old athlete
  5. Calcific AS with anginal chest pain
  6. S4 in a hypertensive patient
  7. Systolic click in 19 year old student with palpitation
  8. S3 gallop in patient with an acute ASMI
  9. MVP with MR in a student with atypical chest pain
  10. Split S1 in a woman with concern about possible MVP
  11. S3 and MR in a patient with CHF due to dilated cardiomyopathy

**5. Self Assessment** (50 min)

- Prove to yourself that you know the basics. Take this self-assessment as often as you like.

**6. Heart Sound and Murmur Bank** (variable)

- Quickly find and listen to 20 different heart sounds by location and type. Use as a pocket reference for future assitance with patients.

---


*Professional Skill Builder: Mastering Cardiac Auscultation in Under Four Hours*, © 2016 by The Regents of the University of Michigan, is licensed under a Creative Commons Attribution-ShareAlike 4.0 International License. More information about the Professional Skill Builder (PSB): psbinformation@umich.edu
